# A Multinational Cost-Consequence Analysis of a Bone Conduction Hearing Implant System—A Randomized Trial of a Conventional vs. a Less Invasive Treatment With New Abutment Technology

**DOI:** 10.3389/fneur.2020.00106

**Published:** 2020-03-13

**Authors:** Marc van Hoof, Stina Wigren, Johan Ivarsson Blechert, Mattias Molin, Henrik Andersson, D. J. M. Mateijsen, Steven J. H. Bom, M. N. Calmels, Antoon J. M. van der Rijt, Mark C. Flynn, Joost van Tongeren, Janny R. Hof, Jan Wouter Brunings, Lucien J. C. Anteunis, Jaime Marco Algarra, Robert Jan Stokroos, Manuela A. Joore

**Affiliations:** ^1^Ear, Nose and Throat (ENT) Department, School for Mental Health and Neuroscience (MHENS), Maastricht University Medical Center, Maastricht, Netherlands; ^2^Cochlear Bone Anchored Solutions AB, Mölnlycke, Sweden; ^3^Statistiska Konsultgruppen, Göteborg, Sweden; ^4^ENT Department, Catharina Hospital, Eindhoven, Netherlands; ^5^ENT Department, Deventer Hospital, Deventer, Netherlands; ^6^ENT Department, Purpan Hospital, Toulouse, France; ^7^ENT Department, Amphia Hospital, Breda, Netherlands; ^8^Research and Innovation, University of Newcastle, Callaghan, NSW, Australia; ^9^Isala Clinics, Department of ENT, Zwolle, Netherlands; ^10^ENT Department, Clinical University Hospital, Valencia, Spain; ^11^Department of Otolaryngology, Head and Neck Surgery, Brain Center Rudolph Magnus, University Medical Center Utrecht, Utrecht, Netherlands; ^12^Department of Clinical Epidemiology and Medical Technology Assessment, Maastricht University Medical Center, Maastricht, Netherlands

**Keywords:** RCT - randomized controlled trial, cost consequence analysis, HTA (health technology assessment), BAHA, bone conducting device, skin integration

## Abstract

**Background:** It is hypothesized that, for patients with hearing loss, surgically placing an implant/abutment combination whilst leaving the subcutaneous tissues intact will improve cosmetic and clinical results, increase quality of life (QoL) for the patient, and reduce medical costs. Here, incremental costs and consequences associated with soft tissue preservation surgery with a hydroxyapatite (HA)-coated abutment (test) were compared with the conventional approach, soft tissue reduction surgery with an all-titanium abutment (control).

**Methods:** A cost-consequence analysis was performed based on data gathered over a period of 3 years in an open randomized (1:1) controlled trial (RCT) running in four European countries (The Netherlands, Spain, France, and Sweden). Subjects with conductive or mixed hearing loss or single-sided sensorineural deafness were included.

**Results:** During the first year, in the Netherlands (NL), France (FR), and Spain (ES) a net cost saving was achieved in favor of the test intervention because of a lower cost associated with surgery time and adverse event treatments [NL €86 (CI −50.33; 219.20), FR €134 (CI −3.63; 261.30), ES €178 (CI 34.12; 97.48)]. In Sweden (SE), the HA-coated abutment was more expensive than the conventional abutment, which neutralized the cost savings and led to a negative cost (SE €-29 CI −160.27; 97.48) of the new treatment modality. After 3 years, the mean cost saving reduced to €17 (CI −191.80; 213.30) in the Netherlands, in Spain to €84.50 (CI −117.90; 289.50), and in France to €80 (CI −99.40; 248.50). The mean additional cost in Sweden increased to €-116 (CI −326.90; 68.10). The consequences in terms of the subjective audiological benefit and Health-related quality of life (HRQoL) were comparable between treatments. A trend was identified for favorable results in the test group for some consequences and statistical significance is achieved for the cosmetic outcome as assessed by the clinician.

**Conclusions:** From this multinational cost-consequence analysis it can be discerned that health care systems can achieve a cost saving during the first year that regresses after 3 years, by implementing soft tissue preservation surgery with a HA-coated abutment in comparison to the conventional treatment. The cosmetic results are better. (sponsored by Cochlear Bone Anchored Solutions AB; Clinical and health economic evaluation with a new Baha® abutment design combined with a minimally invasive surgical technique, ClinicalTrials.gov NCT01796236).

## Introduction

### Hearing Loss and Restoration

Disabling hearing loss (HL) directly affects 360 million people worldwide (1) and is a considerable burden to society and individuals both in terms of health care costs (2, 3) and wellbeing (4). Conventional hearing aids can often restore the ability to communicate and in doing so improve the QoL (5). However, not all types of HL can be effectively dealt with using conventional hearing aids. Wearing conventional hearing aids may lead to severe and chronic ear infections with discharge (6). A semi-implantable hearing device, which is under investigation here, is indicated for those patients who cannot sufficiently benefit from either conventional hearing aids or reconstructive surgery of the ear. Surgical interventions in otology aim to reconstruct the outer or middle ear, but the ability to hear is often only partially restored and relatively high recurrence rates of problems exist (7, 8).

### The Bone Conduction Hearing Implant System

The bone conduction hearing implant (BCHI) system is anchored to the bone behind the ear by means of osseointegration (9, 10). It protrudes through the skin (*percutaneous implant*) using an abutment that connects to the sound processor. This sound processor can be connected at will. The BCHI, in comparison to a conventional hearing aid, converts air-borne sound waves to vibrations of the skull. These vibrations bypass the external and middle ear and reach the inner ear by means of bone conduction. The relative advantage of a *conductive HL* as a result of pathology in the middle or outer ear is that the sensory organ, the inner ear, can be completely intact. If the inner ear also dysfunctions and thus a *sensorineural HL* exists on top of a conductive problem, it is called a *mixed HL*. The BCHI system can still be used in that case, but it is constrained to a certain cochlear reserve or residual sensorineural hearing (11). This type of device does not amplify the sounds much beyond the physiological amplification achieved by the external and middle ear. An advantage of the skull is that it can conduct sound relatively well. Vibrations coming from one side of the head can be transferred with little loss to the other side (12). This makes it possible for patients who only have a one-sided profound deafness [single-sided sensorineural deafness (13) or SSD] to wear the device on the affected side to stimulate the intact inner ear on the other side. This way, sound effectively crosses the skull relieving the “*head shadow effect*” (14). In summary, patients who have pathology in their external or middle ear or have a unilateral deafness prohibiting the use of conventional or CROS hearing aids with sufficient cochlear reserve may often benefit from a bone conduction hearing implant system (BCHI).

With an implantable, skin-breaching (percutaneous) bone conduction hearing implant system, patients have to compromise their bodily integrity in order to restore their hearing. How the appearance and cosmetic outcome [as measured using the Patient and Observer Scar Assessment Scale (POSAS) (15) questionnaire] using this device relate to patients' self-image, wellbeing and QoL, has not yet been investigated in detail. Patients' readiness to make this trade-off and, hence, to accept the intervention (16), might also depend on factors such as culture, perceived benefits, complications, and the acceptance by others. The implant is conventionally placed behind the ear using soft tissue reduction surgery (17), which deliberately removes all subdermal structures. This includes the roots of the hair and effectively creates alopecia around the abutment. Hair plays an important role in masking the system, so this induced baldness may be perceived as problematic. Removing the subdermal structures also leaves a pronounced indentation, removal of skin nerve structures can lead to a loss of sensibility and the formation of scar tissue is frequent (18, 19). Complications such as inflammation (i.e., peri-abutment dermatitis) are burdensome, sometimes painful, and possibly a costly clinical consequence of a percutaneous solution.

### Study Rationale

It is hypothesized that a surgical procedure for placement of the implant/abutment combination preserving the subcutaneous tissues will improve the cosmetic and clinical results and, possibly, also increases the QoL for the patient. Furthermore, it might reduce medical costs, which are associated with the type of intervention. On the other hand, soft tissue reduction had previously been introduced and maintained (17) to *prevent* clinical complications such as inflammation around all-titanium abutments [as measured using the Holgers Index (20)]. This led to the development of a hydroxyapatite (HA)-coated abutment with skin preservation, to prevent these problems and to allow for soft tissue preservation by providing a seal between the abutment and the surrounding tissue which was not possible when using all-titanium abutments with skin reduction (21, 22).

The current cost-consequence analysis (CCA) is part of a *randomized controlled trial* (RCT) which was performed in four European countries of which the clinical results are reported separately (23). The clinical report shows that the primary efficacy outcome difference after 1 year was not statistically significant (29 vs. 13%, *p* = 0.12) in the intention-to-treat (ITT) population. After 3 years, the difference between the two groups had declined and did not reach statistical significance (24 vs. 10%, ITT *p* = 0.45). The recommendation lacked a health economics perspective following the GRADE methodology (24, 25), which evaluates the effect on medical resource use and associated costs. Moreover, the recommendation did not consider possible effects of the less invasive treatment on QoL, audiological benefit or disease-specific health-related QoL in comparison to the conventional treatment. These aspects are investigated here.

This investigation did not collect all relevant resources related to surgery and treatment with a BCHI. Instead, it pragmatically focused on resource use that is affected by the type of intervention (test vs. control treatment). The test treatment was hypothesized to be associated with a reduction in surgical time, a reduction in resources used to treat complications and less frequent hospital visits. The CCA presented here considers the price setting of the different countries. In comparison to other studies, i.e., economic evaluations such as cost-effectiveness analysis (CEA), cost-benefit analysis (CBA), or cost-utility analysis (CUA), CCA allows for a more nuanced, weighted subjective interpretation by the different stakeholders [e.g., policymaker (26), clinician, audiologist]. Because patient preferences and clinical effects of the test intervention prior to this investigation were unclear, a CCA was thought to be the more appropriate approach. Prices and patient preferences for medical interventions also differ between countries (e.g., the percutaneous component's acceptability differs across Europe). Hence, a CCA simplifies international generalizations and interpretations. Therefore, this investigation takes the perspective of every individual country using a scenario analysis.

### Objective of This Study

The objective of this investigation is to compare the incremental costs and consequences associated with *soft tissue preservation surgery with a HA-coated abutment* (test) vs. the conventional approach—*soft tissue reduction surgery with an all-titanium abutment* (control)—for the total patient group and subgroups with different types of HL who were eligible for this system. The time horizon is 1 and 3 years and the analyses are performed from a health care perspective in every participating country.

## Materials and Methods

### Ethics

The final protocol, consent documentation and substantial amendments were approved by the respective ethics committees at each site (De Medisch Ethische Toetsingscommissie (METC), Maastricht, the Netherlands; Regionala Etikprövningsnämnden, Göteborg, Sweden; Comité Ético de Investigación Clínica del Hospital Clínico Universitario de Valencia, Spain; Comité de Protection des Personnes Sud-Ouest et Outre-Mer I/Agence Nationale de Sécurité du Medicament et des Produits de Santé, France). The METC approved the study for all participating Dutch centers; the board of directors of the hospitals subsequently approved conducting this clinical trial according to local legislation. The study was conducted in compliance with the provisions of the Declaration of Helsinki and ISO 14155:2011 “Clinical investigation of medical devices for human subjects—Good clinical practice.” All patients provided written informed consent. The study was registered on ClinicalTrials.gov (NCT01796236).

### Study Design, Treatment, and Oversight

This investigation was designed by the sponsor in conjunction with the authors as a multinational, multi-center, open RCT. Patients were randomly assigned, in a 1:1 ratio, to one of the study treatment arms with site stratification before surgery. The test device was the HA-coated Cochlear™ Baha® BA400 Abutment (length 6, 8, 10, 12, or 14 mm). The control device was the all-titanium Cochlear Baha BA300 Abutment (length 6, 9, or 12 mm). Both abutment types were connected to a Cochlear Baha BI300 Implant. All devices are manufactured by the sponsor, Cochlear Bone Anchored Solutions AB (Mölnlycke, Sweden). In the test group, the surgery consisted of a linear incision *without* soft tissue reduction [as outlined in (19, 23)]. In the control group, surgery was performed using a linear incision with soft tissue reduction. Study visits were performed prior to surgery (baseline), at surgery and 10 days, 3, 6 weeks, 3, 6 months, 1, 2, and 3 years after surgery. The trial was monitored by an independent monitor (A+ Science AB, Stockholm, Sweden) contracted by the sponsor.

The first manuscript draft was written by the first and last author (MvH, MAJ) and subsequently edited by all co-authors. All authors support the reported analyses and subsequent interpretation of the data, which the first and last author validated in detail. All authors vouch for the fidelity of the study to the protocol and supported the decision to submit the manuscript for publication.

### Patient Selection

Adult patients with a conductive or mixed HL or single-sided sensorineural deafness eligible for a bone conduction hearing implant were consecutively enrolled in the study. The trial was conducted in both academic and non-academic hospitals across Europe to reflect clinical practice. The following centers participated in the clinical trial: Maastricht University Medical Center (Maastricht, the Netherlands), Clinical University Hospital Valencia (Valencia, Spain), Purpan Hospital (Toulouse, France), Sahlgrenska University Hospital (Göteborg, Sweden), Amphia Hospital (Breda, the Netherlands), Catharina Hospital (Eindhoven, the Netherlands), Deventer Hospital (Deventer, the Netherlands). Patients were excluded in case of bilateral implantation, uncontrolled diabetes, conditions that could jeopardize osseointegration and/or wound healing, inability to follow the cleaning instructions or to complete study-related questionnaires, concurrent participation in another clinical investigation, insufficient bone quality/quantity during surgery or a condition that may have a substantial impact on the outcome of the investigation as judged by the investigator.

### Outcome Measures

#### Medical Resource Use

Medical resource use was prospectively gathered in the case report forms at every scheduled visit and at extra visits. The following resource items were collected: surgical time (defined as the time between the first incision and the last suture recorded with a stopwatch), the amount of days with an overnight stay at the hospital and the use of different implants and abutments during surgery and thereafter (abutment changes). Resource use as a result of related therapeutic interventions (surgical and pharmaceutics), was also collected. Information about extra visits was gathered (duration, materials used, reason, and health care professionals conducting the visit). Resource use was both split and pooled (scenario analysis) for the different countries for the analysis. Medical resource use was measured in more detail during the first year, which was the period where most visits occur, than over the last 2 years of the study. Some collected treatment end dates were left open during the database lock of the first year (treatment ongoing, or information was missing). Therefore, minor differences might exist in the medical resource consumption as measured during the first year and after the database lock at 3 years.

#### Costs

The costs for different units of medical resource use were identified for every country using publicly or commercially available information (multi-country costing approach). Prices of components related to the primary device under investigation were supplied by Cochlear Bone Anchored Solutions AB. For certain recurring and conventional therapeutic procedures, such as local revision surgery, the consumption of associated medical resources was combined in one unit price. For a full overview of the suppliers of information and the unit costing approach see the supporting information (S1 Supplemental Materials and Methods). Discounting was not performed due to the short timeframe of the investigation.

#### Consequences

Patient reported outcomes were collected using the APHAB (27) for subjective audiological benefit and Health Utilities Index (HUI3) (5, 28) for HRQoL for the unaided (pre-operative visit) and aided (24 weeks, 1 and 3 years post-surgery) condition. The POSAS questionnaire, previously validated for scar assessments (15), was used to assess the investigator's (observer's) and the patient's rating of the appearance of the skin surrounding the abutment. The Holgers index (20) was used to classify any presence of peri-abutment dermatitis (i.e., skin inflammation around the abutment), and was recorded at every post-surgical visit. Sound processor use, defined as the amount of time, estimated by the subject, per day in hours, multiplied by the estimated amount of days, was noted at every follow up visit after sound processor fitting.

#### Statistical Analysis

A power calculation was performed; (23); in summary 100 evaluable patients were considered to be necessary to detect a significant cost saving. Consequences and effect sizes were calculated using the area under the curve (AUC) with linear interpolation to be able to use the three measurement points during the first year (baseline, 6 months, 1 and 3 years) for the APHAB and HUI3. In case of missing data for the calculation of the effect sizes at baseline for the APHAB and HUI3, the mean of similar subjects was imputed. If data were missing at 6 months, the 12-month data of that subject were used. If data were missing at 12 months, the data at 6 months were carried forward. The same procedure was carried out for the 3 years analysis. Descriptive statistics of these outcome values were tabulated without a correction for missing data. The benefit from the intervention was hypothesized to start after sound processor fitting at 6 weeks, as has been presented previously (23), for the APHAB and HUI3. The AUC for the Holgers Index and POSAS were calculated, respectively, at every visit after surgery and at 3 months, 1 and 3 years. Data handling for the Holgers Index has been reported previously (23). In case of missing data for the POSAS at 6 months, the baseline (prior to surgery) was presumed to be 1, and the data at 6 months were interpolated from the data at 12 months. If the data at 1 year were missing, the data from 6 months were carried forward. The same procedure was carried out at 3 years.

The CCA was conducted for predefined subgroups (patients with Conductive HL, Mixed HL and SSD, respectively) and the four countries (The Netherlands, Sweden, Spain and France) using scenario analyses where all individual patients were placed in the price setting of each single country. No corrections for missing resource use and cost data were performed. The average costs per patient per group are reported. In order to obtain 95% credibility intervals (CI) (percentiles) around the average costs and the percentage of cost saving situations, non-parametric bootstrapping (random draws with replacement from the original datasets controlling for test/control and type of hearing loss) with 1,000 iterations was used. The cumulative probability density function for the differences between the groups was calculated per country. All analyses were performed on the ITT population, which included all randomized subjects with at least one follow-up measurement post-surgery. Statistical analyses were performed by independent biostatisticians (Statistiska Konsultgruppen, Göteborg, Sweden) in collaboration with the first (MvH) and last author (MAJ) following a pre-defined statistical analysis plan which was approved by the principal investigator (RS), the responsible statistician (MM) and a sponsor-representative (SW) prior to database lock. No statistical testing was performed because the CCA in the current setup did not lead to a single testable measure of meaning, instead multiple outcomes are presented together with CIs. All analyses were performed using SAS® v9.4 (Cary, United States).

## Results

### Patient Demographics

**Figure 1:**
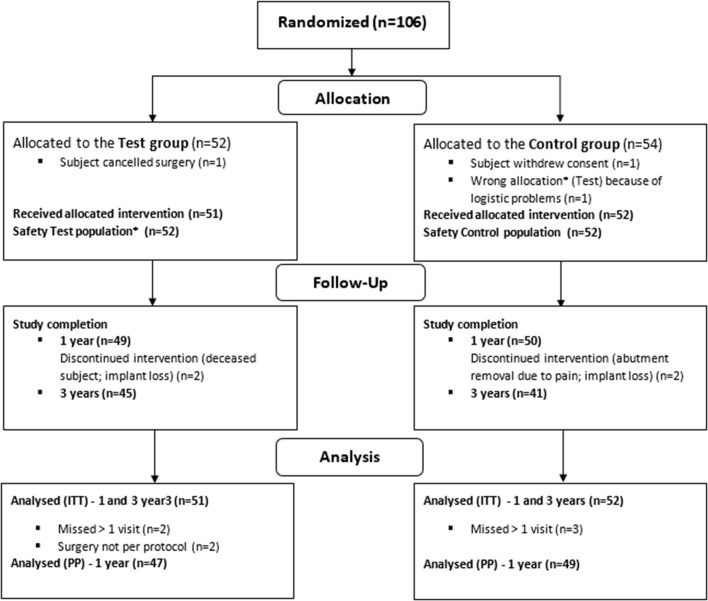
Randomization, treatment and follow-up of subjects. ^*^Due to wrong device allocation in the control group, one subject (randomized to the control group) is considered in the safety population of the test group (23).

In total, 106 patients were enrolled in the clinical trial across seven sites in the four countries. The patients were equally randomized to one of the treatment groups. Of these, 103 patients (51 test, 52 control) were included in the ITT population (Figure 1) (23). The baseline characteristics of the ITT population are shown in Table 1. It can be discerned that the subgroups (per type of HL) are unequally distributed in size.

**Table 1:** Patient demographics.

	**Test group** **(*n* = 51)**	**Control group** **(*n* = 52)**
**Patient demographics**		
Gender, *n* (%)		
Male	26 (51.0%)	25 (48.1%)
Female	25 (49.0%)	27 (51.9%)
Age, mean (SD) years	54.2 (10.9)	51.5 (16.6)
Type of hearing loss, *n* (%)		
Conductive HL	5 (9.8%)	10 (19.2%)
Mixed HL	31 (60.8%)	35 (67.3%)
SSD	15 (29.4%)	7 (13.5%)
Patients per country and site, *n* (%)		
The Netherlands (total)	26 (51.0%)	29 (55.8%)
Maastricht	14 (27.5%)	15 (28.8%)
Breda	6 (11.8%)	8 (15.4%)
Deventer	4 (7.8%)	3 (5.8%)
Eindhoven	2 (3.9%)	3 (5.8%)
Spain		
Valencia	15 (29.4%)	14 (26.9%)
France		
Toulouse	5 (9.8%)	5 (9.6%)
Sweden		
Göteborg	5 (9.8%)	4 (7.7%)

*SSD, Single-sided sensorineural deafness; HL, Hearing loss*.

### Medical Resource Consumption

The medical resource consumption per country is shown in Table 2. During the first year, in all countries, a lower consumption of surgical time can be discerned for the test group. In the Netherlands, patients often underwent surgery using general anesthesia instead of local anesthesia and remained in the hospital after surgery. In Sweden, surgery under general anesthesia was more often performed in the test group. Spain had a relatively high consumption of systemic treatment (such as oral antibiotics) but a lower consumption of pain medication for the treatment of adverse events in comparison to the other countries. The amount of abutment changes was higher in the control group (5) vs. the test group (1). The amount of local revision surgeries (2 vs. 3) and abutment removals was the same (1 vs. 1). The difference between the two groups decreased during the second and third year, mainly due to higher local non-surgical treatment consumption in the test group. This difference is largely due to resource consumption in a single center (Sweden) where 76% of the total resources were consumed by 4 out of 5 local test group subjects, respective to the total test group (*n* = 51). The other three countries maintained a higher rate of resource consumption in the control group, in line with the first-year results. In total 4 abutments were changed in the test group and 5 in the control group. The consumption of pain medication was 1.75 times as high in the control group. A total of 187 and 223 extra visits were conducted during the first year and up to the end of this investigation. Only a fraction of these visits was related to the treatment of adverse events (14 vs. 13%). The reasons for the extra visits are displayed in Table 3.

**Table 2A:** Units per country after 1 year.

**Actual medical resource consumption 1 year**	**Netherlands**		**Sweden**		**Spain**		**France**		**Total**	
	**Test group** **(*n* = 26)**	**Control group** **(*n* = 29)**	**Test group** **(*n* = 5)**	**Control group** **(*n* = 4)**	**Test group** **(*n* = 15)**	**Control group** **(*n* = 14)**	**Test group** **(*n* = 5)**	**Control group** **(*n* = 5)**	**Test group** **(*n* = 51)**	**Control group** **(*n* = 52)**
**PRIMARY SURGERY RELATED UNITS**										
Surgery—local anesthesia (hours)	0.200 (0.200; 0.233) *n* = 6	0.400 (0.383; 0.467) *n* = 5	0.300 (0.200; 0.533) *n* = 3	0.608 (0.475; 0.650) *n* = 4	0.192 (0.167; 0.217) *n* = 12	0.467 (0.400; 0.550) *n* = 13	0.383 (0.333; 0.483) *n* = 5	0.450 (0.417; 0.750) *n* = 3	0.217 (0.183; 0.300) *n* = 26	0.467 (0.400; 0.567) *n* = 25
									**6.70**	**11.85**
Surgery—general anesthesia (hours)	0.225 (0.175; 0.317) *n* = 20	0.292 (0.258; 0.358) *n* = 24	0.350 (0.333; 0.367) *n* = 2		0.167 (0.133; 0.233) *n* = 3	0.717 *n* = 1		0.475 (0.367; 0.583) *n* = 2	0.233 (0.167; 0.317) *n* = 25	0.300 (0.267; 0.400) *n* = 27
									**6.32**	**9.52**
Hospital stay (days)	1.00 (1.00; 1.50) *n* = 20	1.00 (1.00; 1.00) *n* = 26	1.00 (1.00; 1.00) *n* = 2		1.00 (0.00; 1.00) *n* = 3	0.500 (0.00; 1.00) *n* = 2		1.50 (1.00; 2.00) *n* = 2	1.00 (1.00; 1.00) *n* = 25	1.00 (1.00; 1.00) *n* = 30
									**29.00**	**36.00**
Implant/abutment	1.00 (1.00; 1.00) *n* = 26	1.00 (1.00; 1.00) *n* = 29	1.00 (1.00; 1.00) *n* = 5	1.00 (1.00; 1.00) *n* = 4	1.00 (1.00; 1.00) *n* = 15	1.00 (1.00; 1.00) *n* = 14	1.00 (1.00; 1.00) *n* = 5	1.00 (1.00; 1.00) *n* = 5	1.00 (1.00; 1.00) *n* = 51	1.00 (1.00; 1.00) *n* = 52
									**51.00**	**52.00**
**ADVERSE EVENT TREATMENT RELATED UNITS**										
Local non-surgical treatment (combined unit)	15.0 (5.0; 45.0) *n* = 18	34.5 (12.5; 48.5) *n* = 20	233.0 (110.0; 356.0) *n* = 2	22.0 *n* = 1	6.00 (2.00; 7.00) *n* = 5	7.00 (1.0; 44.0) *n* = 3		8.50 (2.00; 15.00) *n* = 2	8.0 (5.0; 45.0) *n* = 25	20.5 (12.0; 44.0) *n* = 26
									**1388.0**	**1315.0**
Systemic treatment (days of use)	10.0 (8.0; 19.0) *n* = 6	15.0 (9.0; 20.0) *n* = 8	11.0 (11.0; 11.0) *n* = 2		8.0 (6.0; 18.0) *n* = 5	7.00 (7.00; 7.00) *n* = 5	18.00 *n* = 1	11.00 *n* = 1	11.0 (8.0; 18.0) *n* = 14	10.5 (7.0; 15.0) *n* = 14
									**472.0**	**237.0**
Pain medication (days of use)	30.0 (19.0; 565.0) *n* = 4	127.0 (57.0; 371.0) *n* = 5	365.0 *n* = 1	307.0 (248.0; 366.0) *n* = 2		6.00 *n* = 1		189.0 *n* = 1	32.0 (28.0; 365.0) *n* = 5	189.0 (57.0; 366.0) *n* = 9
									**1533.0**	**1885.0**
Local revision surgery (combined unit)	1.00 (1.00; 1.00) *n* = 2	2.00 *n* = 1				1.00 *n* = 1			1.00 (1.00; 1.00) *n* = 2	1.50 (1.00; 2.00) *n* = 2
									**2.00**	**3.00**
Diagnostics (X-ray skull, bacterial culture)		2.00 (1.00; 3.00) *n* = 2								2.00 (1.00; 3.00) *n* = 2
										**4.00**
Abutment removals	1.00 *n* = 1	1.00 *n* = 1							1.00 *n* = 1	1.00 *n* = 1
									**1.00**	**1.00**
Abutment changes		1.00 *n* = 1			1.00 *n* = 1	1.00 (1.00; 1.00) *n* = 4			1.000 *n* = 1	1.00 (1.00; 1.00) *n* = 5
										**5.00**
**TIME FOR EXTRA VISITS**										
Audiologist time (hours)	0.750 (0.500; 0.833) *n* = 13	0.750 (0.400; 0.750) *n* = 17	0.500 (0.417; 0.667) *n* = 4	0.583 (0.333; 0.833) *n* = 2	0.500 (0.500; 0.500) *n* = 9	0.500 (0.333; 0.500) *n* = 10		0.750 (0.500; 1.00) *n* = 2	0.500 (0.500; 0.750) *n* = 26	0.500 (0.333; 0.750) *n* = 31
									**16.92**	**18.57**
Surgeon time (hours)	0.85 (0.25; 1.33) *n* = 8	0.37 (0.17; 1.40) *n* = 15	0.542 (0.500; 0.583) *n* = 2	0.500 *n* = 1	0.100 (0.033; 0.167) *n* = 2	0.083 (0.083; 0.533) *n* = 3	0.667 *n* = 1	0.417 (0.333; 0.500) *n* = 2	0.583 (0.167; 0.950) *n* = 13	0.367 (0.167; 0.683) *n* = 21
									**10.82**	**18.37**
Nurse time (hours)		0.917 (0.333; 1.500) *n* = 2	0.250 *n* = 1	0.500 *n* = 1	0.833 (0.542; 1.083) *n* = 4	0.292 (0.167; 0.333) *n* = 6			0.750 (0.333; 0.917) *n* = 5	0.333 (0.250; 0.500) *n* = 9
									**3.50**	**4.00**

*The median number of units with its interquartile range is displayed together with the amount of patients which have incurred this unit in the different countries and for all countries pooled together (Total). A single subject can incur the same treatment multiple times. In the Total column, subtotals per group are marked in bold. The units are quantities unless stated otherwise*.

**Table 2B:** Units per country after 3 years.

**Actual medical resource consumption 3 years**	**Netherlands**		**Sweden**		**Spain**		**France**		**Total**	
	**Test group** **(*n* = 26)**	**Control group** **(*n* = 29)**	**Test group** **(*n* = 5)**	**Control group** **(*n* = 4)**	**Test group** **(*n* = 15)**	**Control group** **(*n* = 14)**	**Test group** **(*n* = 5)**	**Control group** **(*n* = 5)**	**Test group** **(*n* = 51)**	**Control group** **(*n* = 52)**
**ADVERSE EVENT TREATMENT RELATED UNITS**										
Local non-surgical treatment (combined unit)	15.0 (8.0; 29.0) *n* = 18	40.5 (12.5; 53.5) *n* = 20	494 (337; 793) *n* = 4	22.0 *n* = 1	5.00 (3.00; 7.00) *n* = 5	7.00 (1.0; 44.0) *n* = 3		8.50 (2.00; 15.00) *n* = 2	15.0 (6.0; 64.0) *n* = 27	24.5 (12.0; 46.0) *n* = 26
									**2995.0**	**2344.0**
Systemic treatment (days of use)	10.0 (8.0; 23.0) *n* = 8	15.0 (8.0; 15.0) *n* = 9	11.0 (11.0; 11.0) *n* = 2		8.00 (6.00; 8.00) *n* = 5	7.00 (7.00; 11.00) *n* = 6	18.00 *n* = 1	11.00 *n* = 1	10.0 (8.0; 18.0) *n* = 16	10.5 (7.0; 15.0) *n* = 16
									**199.0**	**255.0**
Pain medication (days of use)	32 (28; 380) *n* = 5	899 (57; 1,587) *n* = 5	1,095 *n* = 1	569 (85; 1,053) *n* = 2		6.00 *n* = 1		189.0 *n* = 1	206 (28; 1,095) *n* = 6	189 (57; 1,053) *n* = 9
									**3785.0**	**6608.0**
Local revision surgery (combined unit)	2.50 (2.00; 3.00) *n* = 2	2.00 (2.00; 2.00) *n* = 2				[n] *n* = 1			2.50 (2.00; 3.00) *n* = 2	2.00 (1.00; 2.00) *n* = 3
									**5.00**	**5.00**
Diagnostics (X-ray skull, bacterial culture)		2.00 (1.00; 3.00) *n* = 2								2.00 (1.00; 3.00) *n* = 2
										**4.00**
Abutment removals	1.00 *n* = 1	1.00 *n* = 1							1.00 *n* = 1	1.00 *n* = 1
									**1.00**	**1.00**
Abutment changes	1.00 *n* = 2	1.00 *n* = 2			2.00 *n* = 1	1.00 *n* = 3			1.00 (1.00; 2.00) *n* = 3	1.00 (1.00; 1.00) *n* = 5
									**4.00**	**5.00**
**TIME FOR EXTRA VISITS**										
Audiologist time (hours)	0.750 (0.500; 1.083) *n* = 14	0.750 (0.500; 0.750) *n* = 17	0.500 (0.417; 0.667) *n* = 4	0.583 (0.333; 0.833) *n* = 2	0.500 (0.500; 0.500) *n* = 9	0.500 (0.333; 0.500) *n* = 10		0.750 (0.50; 1.00) *n* = 2	0.500 (0.500; 0.750) *n* = 27	0.500 (0.400; 0.750) *n* = 31
									**18.92**	**19.57**
Surgeon time (hours)	1.05 (0.33; 2.82) *n* = 9	0.37 (0.17; 1.00) *n* = 17	0.500 (0.333; 0.583) *n* = 3	1.00 *n* = 1	0.100 (0.033; 0.167) *n* = 2	0.083 (0.083; 0.617) *n* = 3	0.667 *n* = 1	0.583 (0.333; 0.833) *n* = 2	0.58 (0.17; 1.70) *n* = 15	0.367 (0.167; 1.00) *n* = 23
									**15.93**	**20.45**
Nurse time (hours)		0.917 (0.333; 1.500) *n* = 2	0.250 *n* = 1	1.00 *n* = 1	0.917 (0.542; 1.167) *n* = 4	0.292 (0.167; 0.333) *n* = 6			0.750 (0.333; 1.083) *n* = 5	0.333 (0.250; 0.500) *n* = 9
									**3.67**	**4.50**

**Table 3:** Note that the summed percentage (109.5 and 111%) is higher because some extra visits had more than one indication.

	**Test group (%)**	**Control group (%)**
**REASONS FOR EXTRA VISITS–1 YEAR (*****N*** **=** **187)**		
Adverse event treatment	7.5	15.0
Wound dressing	5.9	3.2
Suture removal	0.5	2.1
After primary surgery		
Sound processor installation	12.8	15.5
Sound processor problem	3.2	4.3
Other	12.8	26.7
**REASONS FOR EXTRA VISITS–3 YEARS (*****N*** **=** **223)**		
Adverse event treatment	14.0	13.1
Wound dressing	5.9	2.7
Suture removal	0.5	1.8
After primary surgery		
Sound processor installation	10.8	13.1
Sound processor problem	3.6	4.5
other	14.9	26.1

### Incurred Costs

The inventory of retrieved prices and an analysis of their reliability are displayed in the supporting information (S2 Supplemental Results). The incurred costs per country are shown in Table 4.

**Table 4A:** Costs per country after 1 year.

**Actual costs per country per patient (€) 1 year**	**Netherlands**		**Sweden**		**Spain**		**France**	
	**Test group** **(*n* = 26)**	**Control group** **(*n* = 29)**	**Test group** **(*n* = 5)**	**Control group** **(*n* = 4)**	**Test group** **(*n* = 15)**	**Control group** **(*n* = 14)**	**Test group** **(*n* = 5)**	**Control group** **(*n* = 5)**
**PRIMARY SURGERY RELATED UNITS**								
Surgery—local anesthesia	25.1 (25.1; 29.3) *n* = 6	50.2 (48.1; 58.6) *n* = 5	23.6 (15.7; 42.0) *n* = 3	47.9 (37.4; 51.2) *n* = 4	18.8 (16.4; 21.3) *n* = 12	45.8 (39.3; 54.0) *n* = 13	44.5 (38.7; 56.2) *n* = 5	52.3 (48.4; 87.1) *n* = 3
Surgery—general anesthesia	144.7 (112.6; 203.7) *n* = 20	187.6 (166.2; 230.5) *n* = 24	209.7 (199.8; 219.7) *n* = 2		110.0 (88.0; 154.0) *n* = 3	473.2 *n* = 1		193.6 (149.5; 237.8) *n* = 2
Hospital stay	275.9 (275.9; 413.9) *n* = 20	275.9 (275.9; 275.9) *n* = 26	247.1 (247.1; 247.1) *n* = 2		210.4 (0.0; 210.4) *n* = 3	105.2 (0.0; 210.4) *n* = 2		637 (425; 849) *n* = 2
Implant/abutment^c^	C+71 (C;C) *n* = 26	C (C;C) *n* = 29	C+180 (C;C) *n* = 5	C (C;C) *n* = 4	C (C;C) *n* = 15	C (C;C) *n* = 14	C (C;C) *n* = 5	C (C;C) *n* = 5
**ADVERSE EVENT TREATMENT**								
Local non-surgical treatment	15.8 (1.4; 128.2) *n* = 18	7.7 (2.9; 102.3) *n* = 20	122.5 (57.8; 187.1) *n* = 2	11.6 *n* = 1	0.67 (0.58; 8.31) *n* = 5	0.58 (0.08; 9.48) *n* = 3		56.4 (1.8; 111.0) *n* = 2
Systemic treatment	14.1 (13.3; 18.2) *n* = 6	24.9 (14.4; 35.2) *n* = 8	16.1 (15.5; 16.7) *n* = 2		1.2 (0.9; 2.6) *n* = 5	1.01 (1.01; 1.09) *n* = 5	14.0 *n* = 1	8.53 *n* = 1
Pain medication	20.4 (12.9; 348.5) *n* = 4	29.3 (17.8; 139.5) *n* = 5	184.1 *n* = 1	341.4 (62.4; 620.3) *n* = 2		1.57 *n* = 1		59.8 *n* = 1
Local revision surgery	90.7 (90.7; 90.7) *n* = 2	181.5 *n* = 1				96.6 *n* = 1		
Diagnostics		93.7 (26.6; 160.8) *n* = 2						
Abutment removals	4.09 *n* = 1	4.09 *n* = 1						
Abutment changes^c^		C *n* = 1			C *n* = 1	C (C;C) *n* = 4		
**TIME FOR EXTRA VISITS**								
Audiologist time	39.3 (26.2; 43.7) *n* = 13	39.3 (21.0; 39.3) *n* = 1 7	12.9 (10.7; 17.2) *n* = 4	15.0 (8.6; 21.4) *n* = 2	17.7 (17.7; 17.7) *n* = 9	17.7 (11.8; 17.7) *n* = 10		29.1 (19.4; 38.8) *n* = 2
Surgeon time	68.2 (20.1; 107.0) *n* = 8	29.4 (13.4; 112.4) *n* = 15	30.0 (27.7; 32.3) *n* = 2	27.7 *n* = 1	6.58 (2.19; 10.96) *n* = 2	5.5 (5.5; 35.1) *n* = 3	50.0 *n* = 1	31.2 (25.0; 37.5) *n* = 2
Nurse time		41.5 (15.1; 67.9) *n* = 2	5.84 *n* = 1	11.7 *n* = 1	27.0 (17.5; 35.1) *n* = 4	9.44 (5.40; 10.79) *n* = 6		

*The median amount of costs in Euro's with its interquartile range is displayed together with the amount of patients. The average is applicable only to those who have incurred this median cost. ^c^Catalog prices were provided by Cochlear Bone Anchored Solutions AB based on a confidentiality agreement for the sole purpose of the calculation and interpretation of the analysis. These prices were censored because of their confidential (trade secret) nature. Incremental differences between interventions (test vs. control) are displayed if applicable*.

**Table 4B:** Costs per country after 3 years.

**Actual costs per country per patient (€) 3 years**	**Netherlands**		**Sweden**		**Spain**		**France**	
	**Test group** **(*n* = 26)**	**Control group** **(*n* = 29)**	**Test group** **(*n* = 5)**	**Control group** **(*n* = 4)**	**Test group** **(*n* = 15)**	**Control group** **(*n* = 14)**	**Test group** **(*n* = 5)**	**Control group** **(*n* = 5)**
**ADVERSE EVENT TREATMENT**								
Local non-surgical treatment	15.8 (43.0; 167.7) *n* = 18	8.0 (0.2; 332.6) *n* = 20	260.8 (139.9; 476.4) *n* = 4	11.6 *n* = 1	0.67 (0.56; 10.63) *n* = 5	0.58 (0.08; 9.48) *n* = 3		56.4 (1.8; 111.0) *n* = 2
Systemic treatment	16.6 (12.1; 33.1) *n* = 8	24.9 (12.5; 42.5) *n* = 9	16.1 (15.5; 16.7) *n* = 2		1.16 (0.54; 5.49) *n* = 5	1.05 (0.96; 12.77) *n* = 6	14.0 *n* = 1	8.53 *n* = 1
Pain medication	21.8 (12.9; 683.0) *n* = 5	203.0 (12.8; 727.4) *n* = 5	552 *n* = 1	204.5 (144.1; 264.8) *n* = 2		1.57 *n* = 1		59.8 *n* = 1
Local revision surgery	226.9 (181.5; 272.2) *n* = 2	181.5 (181.5; 181.5) *n* = 2				96.6 *n* = 1		
Diagnostics		93.7 (26.6; 160.8) *n* = 2						
Abutment removals	4.09 *n* = 1	4.09 *n* = 1						
Abutment changes^c^	C *n* = 2	C *n* = 2			C *n* = 1	C *n* = 3		
**TIME FOR EXTRA VISITS**								
Audiologist time	39.3 (16.6; 122.3) *n* = 14	39.3 (30.2; 47.1) *n* = 17	12.9 (9.7; 19.3) *n* = 4	15.0 (8.6; 21.4) *n* = 2	17.7 (17.7; 17.7) *n* = 9	17.7 (14.2; 17.7) *n* = 10		29.1 (19.4; 38.8) *n* = 2
Surgeon time	84.3 (50.8; 197.2) *n* = 9	29.4 (35.0; 143.5) *n* = 17	27.7 (18.4; 32.3) *n* = 3	55.3 *n* = 1	6.58 (2.19; 10.96) *n* = 2	5.5 (5.5; 40.6) *n* = 3	50.0 *n* = 1	43.7 (25.0; 62.5) *n* = 2
Nurse time		41.5 (15.1; 67.9) *n* = 2	5.84 *n* = 1	23.4 *n* = 1	29.7 (14.2; 40.5) *n* = 4	9.44 (5.40; 12.81) *n* = 6		

**Figure 2:**
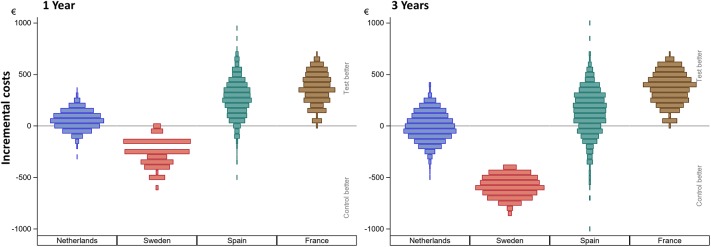
Incurred incremental costs per country after 1 and 3 years. The incremental mean *cost* per subject, per country as simulated using 1,000 bootstraps using unrestricted random sampling which was displayed using a density histogram. A positive number indicates a cost saving in favor for the test intervention. For this simulation all subjects *remained in their country of origin*.

Figure 2 shows the incurred incremental costs per country.

Sweden had higher mean costs in the test group, due to the local practice of more frequent surgery under general anesthesia. Surgery under general anesthesia was performed in the test group in 2 out of 5 patients as opposed to zero in the control group. Also, the test implant and abutments were more expensive (€180 Table 4). In the Netherlands, a mean incremental cost saving was achieved after 1 year, which regressed after 3 years (Figure 2). The test implant and abutments were more expensive (€71—Table 4). There was a cost saving for local and general anesthesia and the hospital stay, a minor cost saving in adverse event treatment costs and extra visits for the first year. Local non-surgical treatment cost, the amount of abutment changes and local revision surgery (relatively) increased for the test group during the last 2 years and resulted in an additional cost after 3 years (Table 4). The test implant and abutments were more expensive (€71—Table 4). In Spain and France, a more pronounced cost saving existed for the test intervention which related to lower adverse event treatment costs (abutment changes) and primary surgery costs during the first year. This difference persisted during the last 2 years.

### Scenario Analysis: Simulation of Costs Pser Country

**Figure 3:**
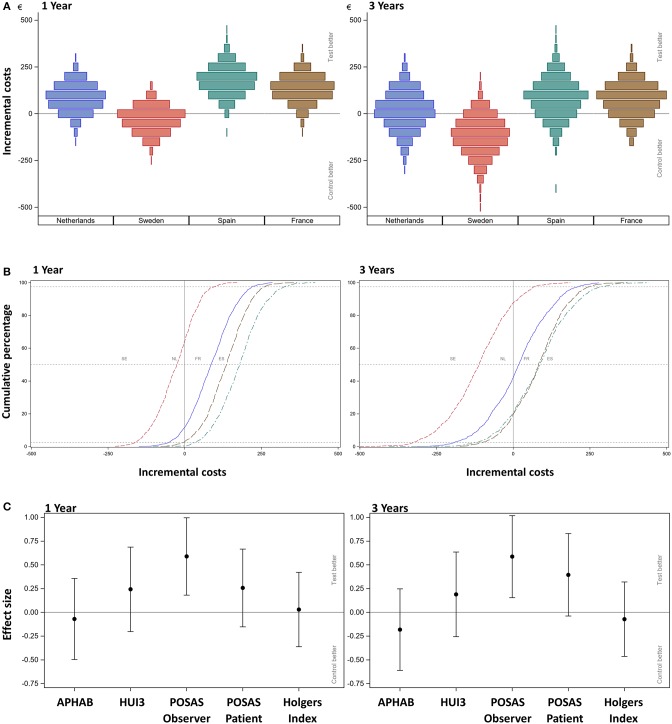
**(A)** Scenario analysis—cost-consequence analysis after 1 and 3 years. The incremental mean *cost* per subject, per country as simulated using 1,000 bootstraps using unrestricted random sampling which was displayed using a density histogram. A positive number indicates a cost saving in favor for the test intervention. For this simulation *all* subjects were *all allocated one-by-one to every of the participating countries per simulation*. **(B)** Scenario analysis after 1 and 3 years. The cumulative probability distribution of incremental costs per country. A positive number indicates a cost saving in favor for the test intervention. The country is indicated per line; notice that the sequence of countries differs from the other figures as the order relates to the increment between the test and control intervention. Dashed lines indicate the 2.5, 50, and 97.5%. **(C)** Cost-consequence analysis after 1 and 3 years. The effect sizes for the different outcome measures (AUCs) over the first year together with the 95% confidence interval are displayed. Note that the APHAB and HUI3 were calculated as the change from (the unaided) baseline.

Figure 3A shows the scenario analyses for the incremental simulated mean cost per patient per country for the first year and after 3 years.

The costs associated with the units per group and per country for the scenario analysis are displayed in Table 5.

**Table 5A:** Scenario analyses: simulation of costs for all patients per country (1 year).

**Simulation of costs for all patients per country (€) 1 year**	**Netherlands**		**Sweden**		**Spain**		**France**	
	**Test group** **(*n* = 51)**	**Control group** **(*n* = 52)**	**Test group** **(*n* = 51)**	**Control group** **(*n* = 52)**	**Test group** **(*n* = 51)**	**Control group** **(*n* = 52)**	**Test group** **(*n* = 51)**	**Control group** **(*n* = 52)**
**PRIMARY SURGERY RELATED UNITS**								
Surgery—local anesthesia	32.3 (14.4) *n* = 26	59.5 (15.4) *n* = 25	20.3 (9.0) *n* = 26	37.3 (9.6) *n* = 25	25.3 (11.3) *n* = 26	46.5 (12.0) 0 *n* = 25	29.9 (13.3) *n* = 26	55.1 (14.2) *n* = 25
Surgery—general anesthesia	162.5 (59.5) *n* = 25	226.7 (89.1) *n* = 27	151.4 (55.4) *n* = 25	211.2 (83.0) *n* = 27	166.8 (61.1) *n* = 25	232.7 (91.5) *n* = 27	103.0 (37.7) *n* = 25	143.7 (56.5) *n* = 27
Hospital stay	320.1 (130.4) *n* = 25	331.1 (133.6) *n* = 30	286.7 (116.8) *n* = 25	296.6 (119.7) *n* = 30	244.1 (99.4) *n* = 25	252.5 (101.9) *n* = 30	492.6 (200.7) *n* = 25	510 (206) *n* = 30
	*156.9*	*191.0*	*140.5*	*171.1*	*119.7*	*145.7*	*241.5*	*294.0*
Implant/abutment^c^	C+71(0) *n* = 51	C(0) *n* = 52	C+180(0) *n* = 51	C(0) *n* = 52	C(0) *n* = 51	C(0) *n* = 52	C(0) *n* = 51	C(0) *n* = 52
**ADVERSE EVENT TREATMENT**								
Local non-surgical treatment	51.3 (72.6) *n* = 25	51.8 (87.8) *n* = 26	65.5 (82.6) *n* = 25	64.1 (88.3) *n* = 26	64.1 (92.1) *n* = 25	65.2 (111.3) *n* = 26	67.1 (90.9) *n* = 25	67.1 (107.1) *n* = 26
	*25.1*	*25.9*	*32.1*	*32.0*	*31.4*	*32.6*	*32.9*	*33.5*
Systemic treatment	28.8 (54.0) *n* = 14	19.1 (21.0) *n* = 14	28.4 (50.7) *n* = 14	21.1 (21.4) *n* = 14	29.8 (82.2) *n* = 14	10.9 (14.6) *n* = 14	26.1 (65.8) *n* = 14	10.9 (12.4) *n* = 14
	*7.91*	*5.14*	*7.79*	*5.69*	*8.17*	*2.95*	*7.16*	*2.95*
Pain medication	175.1 (285.9) *n* = 5	109.1 (137.7) *n* = 9	158.5 (240.5) *n* = 5	131.1 (194.1) *n* = 9	109.7 (185.7) *n* = 5	70.0 (112.7) *n* = 9	98.2 (169.6) *n* = 5	64.9 (77.2) *n* = 9
	*17.2*	*18.9*	*15.5*	*22.7*	*10.8*	*12.1*	*9.62*	*11.2*
Local revision surgery	90.7 (0.0) *n* = 2	136.1 (64.2) *n* = 2	73.1 (0.0) *n* = 2	109.6 (51.7) *n* = 2	96.6 (0.0) *n* = 2	144.9 (68.3) *n* = 2	96.1 (0.0) *n* = 2	144.2 (68.0) *n* = 2
	*3.56*	*5.24*	*2.86*	*4.21*	*3.79*	*5.57*	*3.77*	*5.54*
Diagnostics		93.7 (94.9) *n* = 2		122.2 (135.6) *n* = 2		59.7 (55.0) *n* = 2		74.3 (67.9) *n* = 2
	*0.00*	*3.60*	*0.00*	*4.70*	*0.00*	*2.30*	*0.00*	*2.86*
Abutment removals	4.09 *n* = 1	4.09 *n* = 1	2.82 *n* = 1	2.82 *n* = 1	3.50 *n* = 1	3.50 *n* = 1	3.83 *n* = 1	3.83 *n* = 1
	*0.080*	*0.079*	*0.055*	*0.054*	*0.069*	*0.067*	*0.075*	*0.074*
Abutment changes^c^	C+67 *n* = 1	C(0) *n* = 5	C+181 *n* = 1	C(0) *n* = 5	C *n* = 1	C(0) *n* = 5	C *n* = 1	C(0) *n* = 5
**UNITS RELATED TO EXTRA VISIT DURATION**								
Audiologist time	34.1 (15.6) *n* = 26	31.4 (15.5) *n* = 31	16.7 (7.6) *n* = 26	15.4 (7.6) *n* = 31	23.1 (10.5) *n* = 26	21.2 (10.5) *n* = 31	25.2 (11.5) *n* = 26	23.2 (11.5) *n* = 31
	*17.4*	*18.7*	*8.54*	*9.19*	*11.8*	*12.7*	*12.9*	*13.9*
Surgeon time	66.8 (82.9) *n* = 13	70.2 (106.6) *n* = 21	46.0 (57.1) *n* = 13	48.4 (73.5) *n* = 21	54.7 (67.9) *n* = 13	57.5 (87.4) *n* = 21	62.4 (77.4) *n* = 13	65.6 (99.6) *n* = 21
	*17.0*	*28.3*	*11.7*	*19.5*	*13.9*	*23.2*	*15.9*	*26.5*
Nurse time	31.7 (18.8) *n* = 5	20.1 (19.0) *n* = 9	16.4 (9.7) *n* = 5	10.4 (9.8) *n* = 9	22.7 (13.4) *n* = 5	14.4 (13.6) *n* = 9	28.8 (17.1) *n* = 5	18.3 (17.3) *n* = 9
	*3.11*	*3.48*	*1.60*	*1.80*	*2.22*	*2.49*	*2.83*	*3.17*

*The mean amount of costs in Euro's with its standard deviation is displayed together with the amount of patients which have incurred this cost in the bootstrap simulation. The average is applicable only to those who have incurred this mean cost. The insets in italic do display the mean cost per subject in the group where applicable. (C) Catalog prices were provided by Cochlear Bone Anchored Solutions AB based on a confidentiality agreement for the sole purpose of the calculation and interpretation of the analysis. These prices were censored because of their confidential (trade secret) nature. Incremental differences between interventions (test vs. control) are displayed if applicable*.

**Table 5B:** Scenario analyses: simulation of costs for all patients per country (3 years).

**Simulation of costs for all patients per country (€) 3 years**	**Netherlands**		**Sweden**		**Spain**		**France**	
	**Test group** **(*n* = 51)**	**Control group** **(*n* = 52)**	**Test group** **(*n* = 51)**	**Control group** **(*n* = 52)**	**Test group** **(*n* = 51)**	**Control group** **(*n* = 52)**	**Test group** **(*n* = 51)**	**Control group** **(*n* = 52)**
**ADVERSE EVENT TREATMENT**								
Local non-surgical treatment	83.7 (121.0) *n* = 27	58.9 (91.9) *n* = 26	114.8 (152.1) *n* = 27	84.9 (133.5) *n* = 26	104.9 (153.3) *n* = 27	73.8 (116.0) *n* = 26	111.8 (152.2) *n* = 27	79.7 (120.1) *n* = 26
	*44.32*	*29.45*	*60.78*	*42.45*	*55.54*	*36.9*	*59.19*	*39.85*
Systemic treatment	16.5 (14.0) *n* = 16	18.8 (20.4) *n* = 16	17.2 (13.5) *n* = 16	20.2 (20.6) *n* = 16	8.80 (8.12) *n* = 16	11.1 (14.2) *n* = 16	9.35 (6.52) *n* = 16	10.8 (12.0) *n* = 16
	*5.18*	*5.78*	*5.40*	*6.22*	*2.76*	*3.42*	*9.35*	*3.32*
Pain medication	296.1 (394.4) *n* = 6	238.6 (320.5) *n* = 9	290.8 (378.0) *n* = 6	255.1 (319.1) *n* = 9	134.1 (170.2) *n* = 6	120.5 (148.6) *n* = 9	141.1 (190.4) *n* = 6	150.5 (198.5) *n* = 9
	*34.84*	*41.30*	*34.21*	*44.15*	*15.78*	*20.86*	*16.6*	*26.05*
Local revision surgery	226.9 (64.2) *n* = 2	151.2 (52.4) *n* = 3	182.6 (51.7) *n* = 2	121.8 (42.2) *n* = 3	241.6 (68.3) *n* = 2	161.0 (55.8) *n* = 3	240.3 (68.0) *n* = 2	160.2 (55.5) *n* = 3
	*8.90*	*8.72*	*7.16*	*7.03*	*9.47*	*9.29*	*9.42*	*9.24*
Diagnostics		93.7 (94.9) *n* = 2		122.2 (135.6) *n* = 2		59.7 (55.0) *n* = 2		74.3 (67.9) *n* = 2
	*0.00*	*3.60*	*0.00*	*4.70*	*0.00*	*2.30*	*0.00*	*2.86*
Abutment removals	4.09 *n* = 1	4.09 *n* = 1	2.82 *n* = 1	2.82 *n* = 1	3.50 *n* = 1	3.50 *n* = 1	3.83 *n* = 1	3.83 *n* = 1
	*0.08*	*0.08*	*0.06*	*0.05*	*0.07*	*0.07*	*0.08*	*0.07*
Abutment changes^c^	C+340 (472) *n* = 3	C (0) *n* = 5	C+502 (557) *n* = 3	C (0) *n* = 5	C+385 (667) *n* = 3	C (0) *n* = 5	C+155 (268) *n* = 3	C (0) *n* = 5
**UNITS RELATED TO EXTRA VISIT DURATION**								
Audiologist time	36.7 (21.2) *n* = 27	33.1 (15.9) *n* = 31	18.0 (10.4) *n* = 27	16.2 (7.8) *n* = 31	24.8 (14.4) *n* = 27	22.4 (10.8) *n* = 31	27.2 (15.7) *n* = 27	24.5 (11.8) *n* = 31
	*19.43*	*19.73*	*9.53*	*9.66*	*13.13*	*13.35*	*14.4*	*14.61*
Surgeon time	85.2 (97.4) *n* = 15	71.4 (102.0) *n* = 23	58.8 (67.1) *n* = 1 5	49.2 (70.3) *n* = 23	69.8 (79.8) *n* = 15	58.5 (83.5) *n* = 23	79.6 (91.0) *n* = 27	66.7 (95.3) *n* = 23
	*25.06*	*31.58*	*17.29*	*21.76*	*20.53*	*25.88*	*42.14*	*29.50*
Nurse time	33.2 (20.0) *n* = 5	22.6 (20.7) *n* = 9	17.1 (10.3) *n* = 5	11.7 (10.7) *n* = 9	23.7 (14.3) *n* = 5	16.2 (14.8) *n* = 9	30.2 (18.2) *n* = 5	20.6 (18.9) *n* = 9
	*3.25*	*3.91*	*1.68*	*2.03*	*2.32*	*2.80*	*2.96*	*3.57*

After 1 year, primary surgery related units are associated with the greatest mean cost per subject. The resulting mean *cost saving* in the Netherlands is €86 (CI −50.33; 219.20), in Spain is €178 (CI 34.12; 97.48), and in France is €134 (CI −3.63; 261.30). The mean additional cost in Sweden is €-29 (CI −160.27; 97.48). After 3 years, the resulting mean cost saving in the Netherlands is €17 (CI −191.80; 213.30), in Spain is €84.50 (CI −117.90; 289.50), and in France is €80 (CI −99.40; 248.50). The mean additional cost in Sweden is €-116 (CI −326.90; 68.10). The cumulative probability distributions of the incremental costs per country in Figure 3B show that for the first year, in over 80% of simulations, a net cost saving is achieved in the Netherlands, Spain and France. In the Netherlands the effect has almost neutralized after 3 years. In the majority of simulations, France and Spain continue to achieve a net mean cost saving after 3 years. For Sweden, half of the sigmoid curve is shifted to the left which corresponds to the identified additional costs during the first year. After 3 years, the additional cost has further increased.

### Consequences

The effect sizes for the different outcome measures (Figure 3C) for the total population show that the APHAB has an on average slightly better score in the control group, whose confidence interval includes zero. This outcome remained stable after 3 years.

The absolute APHAB scores are shown in Table 6. The total population at baseline was comparable in regard to the mean global score (test 56.9 SD ± 20.9 vs. control 61.9 SD ± 20.2) but the mean Ease of communication scale (45.5 ± 30.4 vs. 55.4 ± 26.8) was initially better in the test group in comparison to the control group. After 1 year, the absolute scores were within 5 points in both groups for the global score (26.7 ± 13.5 vs. 27.6 ± 12.2) and the Ease of communication scale (17.5 ± 18.0 vs. 19.7 ± 18.7). These scores remained stable over 3 years.

**Table 6A:** Abbreviated profile of hearing aid benefit over the first year.

	**Global**^****LB****^		**Ease of communication**^****LB****^		**Background noise**^****LB****^		**Reverberation**^****LB****^		**Aversiveness**^****LB****^	
	**Test**	**Control**	**Test**	**Control**	**Test**	**Control**	**Test**	**Control**	**Test**	**Control**
**MEAN UNAIDED APHAB—AT BASELINE**										
Total population	56.9 (20.9) *n* = 49	61.9 (20.2) *n* = 48	45.5 (30.4) *n* = 49	55.4 (26.8) *n* = 48	66.6 (18.4) *n* = 49	67.4 (19.1) *n* = 48	58.6 (24.7) *n* = 49	63.0 (25.4) *n* = 48	32.2 (24.6) *n* = 48	25.7 (19.5) *n* = 48
Conductive HL	42.2 (24.6) *n* = 5	64.2 (18.1) *n* = 10	30.3 (37.5) *n* = 5	58.1 (18.8) *n* = 10	64.8 (25.3) *n* = 5	70.4 (19.6) *n* = 10	31.6 (15.7) *n* = 5	64.0 (23.7) *n* = 10	11.0 (10.8) *n* = 5	20.9 (22.1) *n* = 10
Mixed	62.0 (22.7) *n* = 29	65.0 (20.1) *n* = 32	51.9 (32.1) *n* = 29	61.1 (26.3) *n* = 32	68.7 (19.8) *n* = 29	67.1 (19.7) *n* = 32	65.4 (25.6) *n* = 29	66.9 (25.2) *n* = 32	32.6 (27.4) *n* = 28	27.6 (19.7) *n* = 32
SSD	52.0 (12.0) *n* = 15	41.5 (13.4) *n* = 6	38.1 (22.0) *n* = 15	19.9 (9.1) *n* = 6	63.2 (12.9) *n* = 15	63.6 (17.3) *n* = 6	54.5 (18.3) *n* = 15	41.1 (21.3) *n* = 6	38.4 (18.9) *n* = 15	23.5 (15.0) *n* = 6
**MEAN AIDED APHAB—AT 1 YEAR**										
Total population	26.7 (13.5) *n* = 48	27.6 (12.2) *n* = 47	17.5 (18.0) *n* = 47	19.7 (18.7) *n* = 47	29.9 (14.8) *n* = 48	32.3 (14.1) *n* = 47	31.8 (16.5) *n* = 48	30.9 (14.0) *n* = 47	39.3 (23.0) *n* = 47	37.4 (28.8) *n* = 47
Conductive HL	26.3 (13.7) *n* = 5	29.5 (13.3) *n* = 9	16.5 (10.4) *n* = 5	16.6 (21.6) *n* = 9	29.2 (19.4) *n* = 5	38.7 (14.7) *n* = 9	33.2 (15.3) *n* = 5	33.1 (16.3) *n* = 9	46.3 (10.3) *n* = 5	51.1 (22.6) *n* = 9
Mixed HL	22.6 (10.4) *n* = 28	25.5 (11.3) *n* = 32	13.8 (11.1) *n* = 28	20.1 (18.8) *n* = 32	26.7 (14.4) *n* = 28	27.4 (9.6) *n* = 32	27.3 (14.7) *n* = 28	29.1 (12.3) *n* = 32	34.4 (23.2) *n* = 28	33.9 (31.0) *n* = 32
SSD	34.5 (15.8) *n* = 15	36.1 (13.2) *n* = 6	25.3 (27.6) *n* = 14	22.0 (15.8) *n* = 6	36.2 (12.8) *n* = 15	49.1 (18.4) *n* = 6	39.8 (17.8) *n* = 15	37.3 (18.7) *n* = 6	46.6 (24.5) *n* = 14	35.4 (20.6) *n* = 6
	**Global**^**HB**^		**Ease of communication**^**HB**^		**Background noise**^**HB**^		**Reverberation**^**HB**^		**Aversiveness**^**HB**^	
	**Test**	**Control**	**Test**	**Control**	**Test**	**Control**	**Test**	**Control**	**Test**	**Control**
**MEAN AIDED APHAB—BENEFIT FROM UNAIDED AT BASELINE AFTER 1 YEAR**										
Total population	30.6 (23.3) *n* = 47	35.7 (23.3) *n* = 44	28.7 (32.0) *n* = 46	36.5 (31.9) *n* = 44	37.1 (24.1) *n* = 47	36.5 (21.9) *n* = 44	26.3 (26.8) *n* = 47	34.0 (28.1) *n* = 44	−6.71 (28.04) *n* = 45	−12.5 (31.7) *n* = 44
Conductive HL	15.9 (27.8) *n* = 5	37.5 (22.4) *n* = 9	13.8 (31.1) *n* = 5	42.6 (31.9) *n* = 9	35.6 (34.9) *n* = 5	35.4 (17.4) *n* = 9	−1.60 (26.61) *n* = 5	34.5 (25.4) *n* = 9	−35.3 (10.6) *n* = 5	−28.8 (27.3) *n* = 9
Mixed HL	40.6 (22.6) *n* = 26	39.8 (22.0) *n* = 30	41.0 (32.9) *n* = 27	40.9 (30.6) *n* = 30	42.9 (23.0) *n* = 27	40.6 (19.1) *n* = 30	37.9 (24.7) *n* = 27	37.9 (28.6) *n* = 30	−0.308 (26.49) *n* = 26	−7.99 (34.17) *n* = 30
SSD	17.5 (12.8) *n* = 15	7.63 (14.47) *n* = 5	10.5 (18.5) *n* = 14	−1.23 (7.88) *n* = 5	27.1 (20.2) *n* = 15	14.1 (33.6) *n* = 5	14.7 (18.5) *n* = 15	10.0 (20.7) *n* = 5	−8.40 (29.63) *n* = 14	−10.00 (8.00) *n* = 5

*The mean APHAB outcomes with their standard deviations are displayed at baseline (unaided), 1 year (aided) and as the change from baseline (unaided–aided) for the total population and the predefined subgroups. For the absolute scores, a lower number indicates a better score (LB). For the change in the score, a higher number indicates a better score (HB). SSD, single-sided sensorineural deafness. HL, hearing loss*.

**Table 6B:** Abbreviated profile of hearing aid benefit after 3 years.

	**Global**^****LB****^		**Ease of communication**^****LB****^		**Background noise**^****LB****^		**Reverberation**^****LB****^		**Aversiveness**^****LB****^	
	**Test**	**Control**	**Test**	**Control**	**Test**	**Control**	**Test**	**Control**	**Test**	**Control**
**MEAN AIDED APHAB—AT 3 YEARS**										
Total population	28.5 (15.8) *n* = 45	28.1 (14.2) *n* = 41	19.3 (21.3) *n* = 45	18.1 (20.1) *n* = 41	33.5 (16.6) *n* = 45	35.7 (17.2) *n* = 41	32.7 (18.2) *n* = 45	30.6 (13.9) *n* = 41	36.2 (24.8) *n* = 45	36.9 (25.9) *n* = 41
Conductive HL	14.0 (6.1) *n* = 4	19.7 (9.0) *n* = 8	7.88 (5.27) *n* = 4	12.0 (9.8) *n* = 8	15.0 (10.6) *n* = 4	25.0 (14.3) *n* = 8	19.2 (9.9) *n* = 4	22.1 (8.5) *n* = 8	54.0 (16.1) *n* = 4	48.6 (17.0) *n* = 8
Mixed	28.3 (17.3) *n* = 28	28.8 (14.3) *n* = 27	17.7 (21.8) *n* = 28	18.4 (22.2) *n* = 27	34.5 (16.8) *n* = 28	36.6 (17.6) *n* = 27	32.8 (20.3) *n* = 28	31.6 (13.7) *n* = 27	28.0 (24.8) *n* = 28	32.9 (29.2) *n* = 27
SSD	33.4 (11.7) *n* = 13	36.1 (15.6) *n* = 6	26.3 (22.1) *n* = 13	25.0 (20.4) *n* = 6	37.2 (14.7) *n* = 13	46.1 (12.5) *n* = 6	36.7 (13.4) *n* = 13	37.3 (17.4) *n* = 6	48.5 (19.8) *n* = 13	39.5 (14.1) *n* = 6
	**Global**^**HB**^		**Ease of communication**^**HB**^		**Background noise**^**HB**^		**Reverberation**^**HB**^		**Aversiveness**^**HB**^	
	**Test**	**Control**	**Test**	**Control**	**Test**	**Control**	**Test**	**Control**	**Test**	**Control**
**MEAN AIDED APHAB—BENEFIT FROM UNAIDED AT BASELINE AFTER 3 YEARS**										
Total population	29.4 (23.1) *n* = 44	35.5 (22.8) *n* = 38	27.6 (36.6) *n* = 44	38.5 (28.0) *n* = 38	32.8 (21.1) *n* = 44	33.9 (22.2) *n* = 38	27.8 (26.8) *n* = 44	34.2 (30.6) *n* = 38	−2.22 (31.71) *n* = 43	−9.19 (32.95) *n* = 38
Conductive HL	18.6 (15.0) *n* = 4	50.4 (16.1) *n* = 8	5.96 (7.98) *n* = 4	50.3 (16.8) *n* = 8	41.2 (17.5) *n* = 4	52.7 (18.4) *n* = 8	8.54 (22.42) *n* = 4	48.4 (26.6) *n* = 8	−41.2 (22.0) *n* = 4	−24.4 (31.4) *n* = 8
Mixed HL	35.5 (25.3) *n* = 27	36.0 (21.4) *n* = 25	37.5 (39.7) *n* = 27	42.3 (27.7) *n* = 25	35.2 (22.0) *n* = 27	30.7 (19.0) *n* = 25	33.9 (28.6) *n* = 27	35.0 (30.4) *n* = 25	7.52 (28.27) *n* = 26	−3.25 (34.73) *n* = 25
SSD	20.1 (15.6) *n* = 13	9.56 (18.04) *n* = 5	13.9 (28.2) *n* = 13	0.97 (6.65) *n* = 5	25.2 (19.1) *n* = 13	19.8 (28.0) *n* = 5	21.2 (20.6) *n* = 13	7.90 (24.8) *n* = 5	−9.72 (30.96) *n* = 13	−14.6 (19.0) *n* = 5

The overall HRQoL as measured using the HUI3 shows a small advantage for the test group (Figure 3C). This confidence interval does not exceed zero. The mean health-related HRQoL (Table 7) at baseline was similar (0.62 ± 0.27 vs. 0.62 ± 0.26).

**Table 7A:** Quality of Life measure over the first year.

	**Health-related QoL**		Single relevant attribute scores^*^							
			**Hearing**		**Emotion**		**Pain**		**Speech**	
	**Test**	**Control**	**Test**	**Control**	**Test**	**Control**	**Test**	**Control**	**Test**	**Control**
**MEAN HUI3—AT BASELINE**										
Total population	0.617 (0.272) *n* = 45	0.620 (0.262) *n* = 44	0.627 (0.304) *n* = 49	0.601 (0.304) *n* = 47	0.893 (0.170) *n* = 50	0.945 (0.108) *n* = 51	0.901 (0.174) *n* = 50	0.918 (0.164) *n* = 51	0.913 (0.165) *n* = 50	0.906 (0.165) *n* = 50
Conductive HL	0.777 (0.204) *n* = 5	0.659 (0.260) *n* = 10	0.734 (0.260) *n* = 5	0.605 (0.293) *n* = 10	0.946 (0.121) *n* = 5	0.973 (0.043) *n* = 10	0.968 (0.044) *n* = 5	0.853 (0.308) *n* = 10	1.00 (0.00) *n* = 5	0.934 (0.139) *n* = 10
Mixed HL	0.531 (0.285) *n* = 25	0.552 (0.260) *n* = 27	0.579 (0.330) *n* = 29	0.556 (0.313) *n* = 30	0.843 (0.198) *n* = 30	0.933 (0.128) *n* = 34	0.891 (0.197) *n* = 30	0.925 (0.112) *n* = 34	0.897 (0.169) *n* = 30	0.878 (0.182) *n* = 33
SSD	0.707 (0.225) *n* = 15	0.828 (0.145) *n* = 7	0.683 (0.261) *n* = 15	0.790 (0.237) *n* = 7	0.976 (0.041) *n* = 15	0.961 (0.048) *n* = 7	0.899 (0.155) *n* = 15	0.977 (0.039) *n* = 7	0.917 (0.182) *n* = 15	1.00 (0.00) *n* = 7
**MEAN HUI3—AT 1 YEAR**										
Total population	0.688 (0.206) *n* = 48	0.650 (0.232) *n* = 47	0.726 (0.152) *n* = 48	0.760 (0.083) *n* = 48	0.911 (0.125) *n* = 48	0.900 (0.160) *n* = 48	0.922 (0.180) *n* = 48	0.899 (0.215) *n* = 48	0.975 (0.078) *n* = 48	0.975 (0.078) *n* = 48
Conductive HL	0.861 (0.058) *n* = 5	0.700 (0.195) *n* = 9	0.800 (0.082) *n* = 5	0.792 (0.107) *n* = 9	0.982 (0.040) *n* = 5	0.910 (0.110) *n* = 9	0.984 (0.036) *n* = 5	0.889 (0.157) *n* = 9	1.00 (0.00) *n* = 5	0.980 (0.06) *n* = 9
Mixed HL	0.624 (0.217) *n* = 28	0.614 (0.250) *n* = 32	0.687 (0.147) *n* = 28	0.751 (0.076) *n* = 33	0.857 (0.138) *n* = 28	0.885 (0.182) *n* = 33	0.925 (0.196) *n* = 28	0.892 (0.245) *n* = 33	0.982 (0.070) *n* = 28	0.975 (0.085) *n* = 33
SSD	0.749 (0.165) *n* = 15	0.763 (0.137) *n* = 6	0.773 (0.163) *n* = 15	0.760 (0.077) *n* = 6	0.988 (0.032) *n* = 15	0.970 (0.046) *n* = 6	0.894 (0.179) *n* = 15	0.948 (0.093) *n* = 6	0.954 (0.101) *n* = 15	0.970 (0.073) *n* = 6
**HUI3—CHANGE FROM BASELINE AFTER 1 YEAR**										
Total population	0.048 (0.245) *n* = 43	0.029 (0.286) *n* = 41	0.080 (0.311) *n* = 47	0.179 (0.342) *n* = 45	0.003 (0.167) *n* = 48	−0.039 (0.189) *n* = 48	0.004 (0.167) *n* = 48	−0.017 (0.260) *n* = 48	0.058 (0.160) *n* = 48	0.060 (0.182) *n* = 47
Conductive HL	0.084 (0.185) *n* = 5	0.045 (0.318) *n* = 9	0.066 (0.232) *n* = 5	0.231 (0.286) *n* = 9	0.036 (0.136) *n* = 5	−0.070 (0.117) *n* = 9	0.016 (0.067) *n* = 5	0.052 (0.376) *n* = 9	0.00 (0.00) *n* = 5	0.017 (0.05) *n* = 9
Mixed HL	0.045 (0.284) *n* = 23	0.054 (0.306) *n* = 26	0.077 (0.343) *n* = 27	0.215 (0.364) *n* = 30	−0.008 (0.210) *n* = 28	−0.037 (0.219) *n* = 33	0.006 (0.171) *n* = 28	−0.035 (0.247) *n* = 33	0.080 (0.170) *n* = 28	0.089 (0.211) *n* = 32
SSD	0.042 (0.209) *n* = 15	−0.101 (0.052) *n* = 6	0.089 (0.291) *n* = 15	−0.082 (0.178) *n* = 6	0.012 (0.046) *n* = 15	0.00 (0.057) *n* = 6	−0.005 (0.191) *n* = 15	−0.025 (0.079) *n* = 6	0.037 (0.165) *n* = 15	−0.030 (0.073) *n* = 6

*The mean HUI3 outcomes with their standard deviations are displayed at baseline, 1 year and as the change from baseline for the total population and the predefined subgroups*.

* *Including the previously identified attributes (29) with the addition of pain because of the surgical intervention (23). QoL Quality of Life. SSD, Single-sided sensorineural deafness. HL, Hearing loss*.

**Table 7B:** Quality of Life measure after 3 years.

	**Health-related QoL**		Single relevant attribute scores*							
			**Hearing**		**Emotion**		**Pain**		**Speech**	
	**Test**	**Control**	**Test**	**Control**	**Test**	**Control**	**Test**	**Control**	**Test**	**Control**
**MEAN HUI3—AT BASELINE**										
Total population	0.758 (0.147) *n* = 43	0.639 (0.239) *n* = 38	0.728 (0.172) *n* = 45	0.683 (0.197) *n* = 41	0.950 (0.073) *n* = 45	0.906 (0.155) *n* = 41	0.977 (0.054) *n* = 45	0.926 (0.142) *n* = 41	0.984 (0.062) *n* = 44	0.956 (0.127) *n* = 40
Conductive HL	0.875 (0.084) *n* = 4	0.676 (0.118) *n* = 8	0.783 (0.145) *n* = 4	0.748 (0.069) *n* = 8	1.000 (0.000) *n* = 4	0.966 (0.047) *n* = 8	1.00 (0.00) *n* = 4	0.886 (0.182) *n* = 8	1.00 (0.00) *n* = 4	0.959 (0.117) *n* = 8
Mixed HL	0.717 (0.161) *n* = 27	0.583 (0.269) *n* = 24	0.700 (0.191) *n* = 28	0.639 (0.211 *n* = 27	0.923 (0.080) *n* = 28	0.870 (0.179) *n* = 27	0.978 (0.051) *n* = 28	0.927 (0.144) *n* = 27	0.982 (0.070) *n* = 28	0.945 (0.144) *n* = 26
SSD	0.814 (0.086) *n* = 12	0.814 (0.137) *n* = 6	0.772 (0.129) *n* = 13	0.793 (0.201) *n* = 6	0.993 (0.025) *n* = 13	0.985 (0.037) *n* = 6	0.970 (0.067) *n* = 13	0.973 (0.041) *n* = 6	0.985 (0.052) *n* = 12	1.00 (0.00) *n* = 6
**HUI3—CHANGE FROM BASELINE AFTER 3 YEARS**										
Total population	0.114 (0.254) *n* = 38	0.026 (0.300) *n* = 31	0.072 (0.344) *n* = 44	0.049 (0.340) *n* = 37	0.040 (0.148) *n* = 45	−0.030 (0.156) *n* = 40	0.058 (0.119) *n* = 45	0.018 (0.230) *n* = 40	0.075 (0.181) *n* = 44	0.060 (0.172) *n* = 38
Conductive HL	0.035 (0.140) *n* = 4	0.030 (0.276) *n* = 8	0.043 (0.247) *n* = 4	0.176 (0.274) *n* = 8	0.00 (0.00) *n* = 4	−0.011 (0.032) *n* = 8	0.020 (0.040) *n* = 4	0.070 (0.408) *n* = 8	0.00 (0.00) *n* = 4	0.00 (0.176) *n* = 8
Mixed HL	0.129 (0.278) *n* = 22	0.050 (0.362) *n* = 17	0.084 (0.384) *n* = 27	0.030 (0.388) *n* = 23	0.058 (0.184) *n* = 28	−0.047 (0.191) *n* = 26	0.058 (0.107) *n* = 28	0.007 (0.183) *n* = 26	0.080 (0.180) *n* = 28	0.095 (0.186) *n* = 24
SSD	0.113 (0.249) *n* = 12	−0.049 (0.062) *n* = 6	0.058 (0.298) *n* = 13	−0.048 (0.164) *n* = 6	0.014 (0.050) *n* = 13	0.015 (0.037) *n* = 6	0.069 (0.157) *n* = 13	0.00 (0.072) *n* = 6	0.089 (0.214) *n* = 12	0.00 (0.00) *n* = 6

One year after the intervention, the scores increased (0.05 ± 0.25) in the test group and control group (0.03 ± 0.29). Upon assessment of the change in single relevant attribute scores Hearing, Emotion and Pain over the first year, it can be discerned that the control group achieves a larger increase than the test group on the hearing attribute but attains a decrease on the emotion and pain score. 21% of subjects scored the highest level at baseline for the hearing attribute while 4% of the entire study population (*n* = 103) did after 1 year. After 3 years, the scores increased (0.11 ± 0.25) in the test group in comparison to the control group (0.03 ± 0.30). The Hearing attribute remained largely the same. However, the Pain and Emotion attributes showed an increased benefit in the test group.

The cosmetic outcomes as measured with the POSAS questionnaire shows an effect size for the evaluation of the observer that exceeds zero in favor of the test treatment after 1 and 3 years. The POSAS questionnaire as completed by the patient also shows a small overall positive effect. This effect does not exceed zero after 1 year, but does after 3 years. Inflammation as measured using the Holgers Index is approximately equal in both groups. After 3 years these effects were similar.

Sound processor use per subgroup is displayed in Table 8.

**Table 8:** The sound processor use at 1 and 3 years is tabulated for the different subgroups.

	**Hours per week mean (SD)**	**Non-use**
**SOUND PROCESSOR USE AT 1 YEAR**		
Conductive HL (*n* = 14)	79.4 (40.7)	1 (7.1%)
Mixed HL (*n* = 61)	78.0 (29.5)	1 (1.6%)
SSD (*n* = 21)	71.5 (35.4)	1 (4.8%)
**SOUND PROCESSOR USE AT 3 YEARS**		
Conductive HL (*n* = 12)	84.9 (33.0)	0 (0.0%)
Mixed HL (*n* = 55)	76.1 (31.2)	1 (1.8%)
SSD (*n* = 19)	69.8 (35.1)	0 (0.0%)

*SSD, Single-sided sensorineural deafness. HL, Hearing loss*.

### Subgroup Analysis Based on the Type of HL

**Figure 4:**
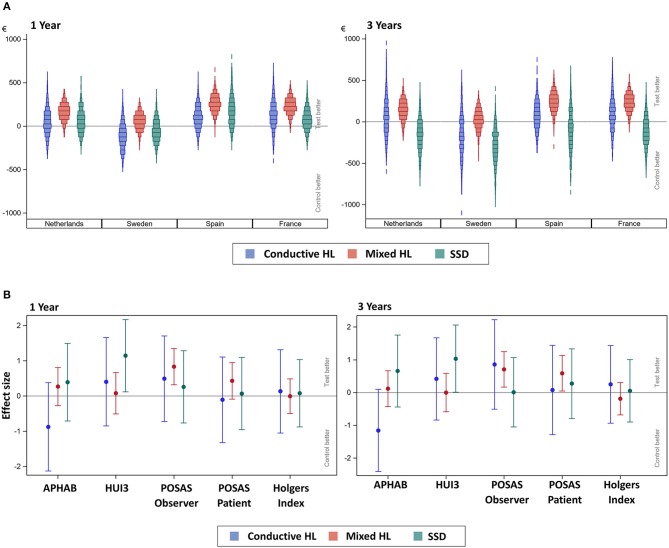
**(A)** Scenario analysis—subgroup cost-consequence analysis after 1 and 3 years. The incremental mean cost per subject, per country, per subgroup as simulated using 1,000 bootstraps using unrestricted random sampling which was displayed using a density histogram. A positive number indicates a cost saving in favor for the test intervention. For this simulation *all subjects per subgroup were allocated one-by-one to every of the participating countries per simulation*. SSD, single-sided sensorineural deafness. **(B)** Subgroup cost-consequence analysis after 1 and 3 years. The effect sizes for the different outcome measures (AUCs) over the first year together with the 95% confidence interval are displayed. Note that the APHAB and HUI3 were calculated as the change from (the unaided) baseline.

The subgroup scenario CCA (Figure 4A) shows that the subgroups have a different distribution of costs.

During the first year, the median cost-saving is greatest and above the zero-difference line in every country for the Mixed HL subgroup, which also shows a similar distribution in every country. The costs for the Conductive HL and SSD subgroups are more variable and do not follow a normal distribution. After 3 years, the distributions of the Conductive HL and SSD subgroups shifted downwards, resulting in an additional cost for the test group. The Mixed HL subgroup achieved a similar cost saving in the test group.

The differences in outcome measures for the subgroups when comparing the test vs. the control intervention subgroups over the first year and after 3 years (effect sizes) are displayed in Figure 4B.

During the first year, the direction of the effect size (sign of the mean) in the subgroups is similar or equal to the total population (Figure 3C) with the exception of the APHAB score. In the APHAB, the Conductive HL group achieves a better score in the control group, while the Mixed HL and SSD group achieves a better score in the test group. Both confidence intervals do not exceed zero. The SSD group achieves a better score with the test intervention for the HUI3 outcome measure, which does exceed zero. This effect persists after 3 years.

The APHAB at baseline shows a large difference between subgroups for the Global score with a similar trend as the Ease of communication scale (Table 6). The group with SSD has the lowest hearing impairment as measured using the APHAB. There is a difference in scores between the test and control group which is influenced by the high inequality in the number of patients. The Global and Ease of communication scores at 1 year have become more equal in comparison to the baseline situation. The subgroup with SSD has a higher impairment than the Conductive and Mixed HL subgroups at 1 year in contrast to the baseline situation, as reflected by the Global and Ease of communication scores. This corresponds to a higher benefit after 1 year for the Conductive and Mixed HL subgroups. Effects at 3 years were similar.

The mean health-related QoL (Table 7) shows that the mean scores at baseline, at 1 and after 3 years differ per type of subgroup. The change from baseline does not differ much per subgroup.

## Discussion

The objective of this study was to investigate the costs and consequences (subjective audiological outcome and health-related QoL) over the first year and after 3 years, of a HA-coated abutment placed using soft tissue preservation surgery compared to a conventional titanium abutment with soft tissue reduction surgery. In regard to the cost differences, it has been shown that within the different European countries different price settings exist which influenced the cost difference between the two interventions. During the first year, in the Netherlands, France and Spain a net cost saving was achieved in favor of soft tissue preservation with a HA-coated abutment because of a lower unit cost associated with surgery time and adverse event treatments [NL €86 (CI −50.33; 219.20), FR €134 (CI −3.63; 261.30), ES €178 (CI 34.12; 97.48)]. In Sweden, the HA-coated abutment was more expensive than the conventional abutment, which contributed to neutralize the cost savings and led to a small negative cost (SE €-29 CI −160.27; 97.48) of the new treatment modality in Sweden. After 3 years, the resulting mean cost saving reduced to €17 (CI −191.80; 213.30) in the Netherlands, in Spain to €84.50 (CI −117.90; 289.50) and in France to €80 (CI −99.40; 248.50). The mean additional cost in Sweden increased to €-116 (CI −326.90; 68.10).

The consequences in terms of the subjective audiological benefit and HRQoL are comparable between the two interventions. The mean HRQoL shows that the total population at baseline is almost equal (test 0.62 SD ± 0.27 vs. control 0.62 SD ± 0.26), but low in comparison to an age matched reference population from the USA (30) (0.78 95% confidence interval 0.76; 0.80). One year after the intervention, mean HRQoL scores increased with 0.05 ± 0.25 in the test group vs. 0.03 ± 0.29 in the control group. Three years after the intervention, these scores increased further for the test group 0.11 ± 0.25, whereas the control group remained stable at 0.03 ± 0.30. A change in score of 0.05 is generally considered meaningful (31). A trend exists for favorable results in the HA-coated abutment group for some consequences but statistical significance is only achieved for the cosmetic outcome as assessed by the clinician after 1 and 3 years. In the previous clinical publication of this trial (23) improvements of clinical outcomes such as a reduction of numbness and pain around the implant have been identified.

### Recommendation and Implementation

Based on these results, if there is no or a minor additional cost associated with the implant and HA-coated abutment, it can be assumed that there are health care cost savings during the first year by implementing the test intervention. No significant additional efforts or costs were identified for the implementation of the new intervention except for the purchase of an autoclavable surgical instrument (skin thickness ruler). Uncertainty analysis showed that the HA-coated abutment has a probability of over 80% for being cost saving. This effect was no longer present after 3 years. The patient preferences for this new intervention have not been formally investigated, however it can be expected that the clinical benefits which have been identified in this trial (23) make this system equally or even more acceptable to patients. Hence, from a patient, clinician and policy maker perspective (24, 25) we recommend the use of soft tissue preservation with a HA-coated abutment over soft tissue reduction with a titanium abutment. However, it cannot be assessed if the improvements stem from the change in abutment and/or surgical procedure (23).

Many health technology assessments based on multi-country study data, assume that the price setting in one country does not differ considerably from other comparable countries. Approximately half of multinational economic evaluations used only one country for unit costing (32). However, as can be seen in this trial, differences between countries in price settings can be large enough to neutralize cost savings which might be achieved from clinical improvements.

The representability of the selected countries and the consistency of findings in this investigation indicate that the outcome generalizability can be expected to be sufficient to extrapolate recommendations to other high-income countries in Europe.

### Patient HL, Use Patterns and Expected Benefits

The results of the subgroup analysis based on the type of HL could have been biased due to differences in baseline characteristics between the less invasive treatment and conventional treatment groups, despite randomization. This bias might have been exaggerated by the non-linearity in the questionnaire scoring system. Another factor which might play a role in the results of the subgroup analyses, is that soft tissue preservation influences sound transduction. Although the SSD patients had the lowest hearing impairment according to the APHAB scores at baseline in comparison to the other subgroups, they had the highest hearing impairment and smallest decrease in impairment after 1 and 3 years of treatment with the BCHI. This probably relates to the fact that the subjects in this HL group cannot benefit from binaural hearing, whilst the other two subgroups can. Although the QoL benefit in this subgroup did not differ between the interventions, the results found here do correspond to APHAB outcomes previously presented (33). This remains to be a point of attention in expectation management, future evaluation, and patient counseling (34). Another finding was that the cost difference observed during the first year in the different subgroups might differ. A possible explanation is that patients with a conductive and mixed HL might visit the outpatient clinic more frequently for follow up on their associated middle ear complaints. As it is customary to do an additional check up on the BCHI, this might result in a different resource use and cost profile.

The BCHI is a medical device with a different function for people with different kinds of HL. The questionnaires that are currently most commonly used only address a part of these functional situations (e.g., being able to hear others speak). Patients with SSD for example value the situational awareness which is increased as the device alleviates the head shadow effect [amongst other effects (14)]. This allows them to effectively change the way they interact with people as they are able to follow conversations better in noisy backgrounds and become aware of sounds at the side of their deafness. Questions have been raised that the subgroup with SSD might be a *concern* in terms of use frequency or duration, especially in the long term (35). Patients with SSD do not always wear the device the whole day, but often selectively use the device when needed (36). This explains the lower sound processor use for this group as found here. The focus in the current literature is often to maximize the number of hours of use, as it is assumed to reflect the effectiveness of the treatment and it has also been related to economic effectiveness (37). Although absolute non-use is an important and potentially costly point to address, which was low in this trial, this association can be questioned. As the benefits of the device and the number of hours of use are not necessarily linearly related, it might not be as straightforward as to conclude that fewer hours of use is a worse result. The relative higher benefit in terms of QoL for the SSD less invasive group also suggests that these patients might value aspects differently. For example, patients who are not being able to hear with a bilateral conductive HL might value complications such as pain and numbness, which differs between the conventional treatment and less invasive treatment, differently than patients with SSD who have a disability only in certain situations. Furthermore, the real reasons for non- and decreased use (e.g., the dependency on the device) are important to consider over the longer term. These cannot be expected to be easily identified using a questionnaire and should be subject to further (qualitative) study as patients might not be aware of the reason themselves. The way in which these patients benefit from the device over time is also important to study and account for in a lifetime costing and benefit horizon in a full economic evaluation.

The costs for adverse event treatment was lower in this study in comparison to previous HTA reports (37). One of the factors causing this is that implant extrusions were not accounted for. Although it can be expected, following the cost split up by Colquitt et al. (37), that the total costs related to the treatment of adverse events is lower for soft tissue preservation with a HA-abutment than what has been previously modeled. Additionally, the expectation is that the trend showing declining complications over time, which was identified in the clinical trial (23), will also lead to a reduction in adverse event treatment costs over time.

The cost-effectiveness of the BCHI has been previously questioned (37, 38). However, as discussed above, the type of HL and corresponding benefits from the device can strongly differ between subgroups of eligible patients (39). This begs the question if such an investigation should not better focus on determining the cost-effectiveness of treatment protocols (*clinical strategies*) which include the device under consideration in this investigation, and its direct alternatives, for certain specific types of patients. This could then also consider the different available devices on the market (40), the preferences of the clinician and the patient in a decision tree resulting from it. This way, specific situations or contra-indications such as having chronic otorrhea (“*running ears*”) from conventional hearing aids (41) can also be considered in cost-effectiveness. Transcutaneous systems should also be incorporated in this decision tree. These devices are completely concealable (*no* skin penetration) when not in active use and they possibly have lower complication rates as well. A disadvantage is that passive transcutaneous devices often feature smaller sound amplification benefits and require relatively more invasive surgery (40). Discrete-Choice Experiments can additionally be used to consider the patient preferences (42) for the different options available in the branches of the treatment protocol specific for patient subgroups, which also includes non-invasive options such as the CROS hearing aid.

For this evaluation other benefits which have not been measured before could also be considered. The end goal of most interventions for hearing restoration is to “*hear again*.” But, except for the ability to perceive sound, this often comes down to increasing the ability to interact socially with people, to be able to achieve at work or in school and enjoy life. This is more or less true for all subgroups of BCHI users. In that sense, the capability approach as advocated by Nussbaum and Sen (43) which was incorporated in a validated questionnaire by Coast et al. [ICECAP (44)] might be a good additional outcome to consider for future investigations into (cost)effectiveness.

## Limitations

Many possibly relevant costs have been disregarded and not measured in this study [e.g., productivity costs, sound processor costs (45), out-of-pocket payments such as batteries and insurances etc.]. The focus was on health care costs which showed a possible difference between treatment interventions and countries. However, the costs which have been accounted for can be used for future cost-effectiveness models. Furthermore, this does not allow for a calculation of the total difference in costs.

One of the assumptions in this study was that patients have equal risks of complications in different countries and that clinicians practice medicine similarly in different countries. The first is unknown and the latter is known not to be the case from data presented here and in the literature (46). However, the small sample size in this study inhibits making this an object of study as well. The subgroups in this study are small and relatively unequal. In the future, trials should consider stratifying the randomization process based on the type of HL of the patients.

The case report forms of this study were not suitable for determining the exact costs of an implant loss. Although it is an infrequent complication, the associated costs can be considerable and also depend on the decision of the patient to have a re-implantation. Future studies should include these costs.

As mentioned previously (23), the POSAS questionnaire lacks sensitivity and, hence, suitability for measuring the cosmetic outcome in BCHI surgery, as it does not relate to the differences the intervention affects (e.g., local alopecia, indentation etc.). The questionnaires HUI3 and APHAB are frequently used in BCHI related research. HUI3 represents general health related QoL and has been found to be sensitive to changes related to hearing loss (5, 29). Previous investigations have shown no to small meaningful changes (37, 47) depending on the type of HL after the BCHI intervention. The framing of the hearing related question is clearly not covering the full bandwidth of possible types of HL, the effect of rehabilitation and associated disabilities. The interpretation of the hearing question in the HUI3 can lead to a lower QoL score with the BCHI. In this investigation a contradiction was observed; 21% of subjects scored the maximum level at baseline for the hearing attribute *without* the sound processor, whereas 4% of the entire study population did after 1 year *with* the sound processor. However, at this point in time, there exists no other, more suitable questionnaire. The APHAB is conceptually intended to reflect the disability with everyday listening situations for the hearing impaired and to provide a measure of changes achieved with hearing aid fittings (27). It does not contain any social or emotional dimensions other than what is implied by the amount of disability. Moreover, the (linear) composition and weighing of the individual questions in summing the total score inhibits an assessment of reliable total hearing related disability. It does not scale the severity of the hearing loss, for example using “deaf” and “perfect sense of hearing” as the anchors (0–1) (48). Indeed, a hearing related health state approach for a questionnaire would be more appropriate and has been previously proposed (49).

## Conclusion

From this multinational cost-consequence analysis, performed with data gathered in a clinical RCT, it can be discerned that health care systems can achieve a cost saving during the first year that regresses after 3 years, by implementing soft tissue preservation surgery with a HA-coated abutment in comparison to the conventional treatment (soft tissue reduction with a titanium abutment). This is applicable as long as there exists no large additional cost difference between the two titanium implant/abutment combinations. The consequences of the new intervention are similar to conventional treatment, and cosmetic results are better.

## Data Availability Statement

The datasets generated for this study are available on request to the corresponding author.

## Ethics Statement

The final protocol, consent documentation and substantial amendments were approved by the respective ethics committees at each site [De Medisch Ethische Toetsingscommissie (METC), Maastricht, the Netherlands; Regionala Etikprövningsnämnden, Göteborg, Sweden; Comité Ético de Investigación Clínica del Hospital Clínico Universitario de Valencia, Spain; Comité de Protection des Personnes Sud-Ouest et Outre-Mer I/Agence Nationale de Sécurité du Medicament et des Produits de Santé, France). The METC approved the study for all participating Dutch centers; the board of directors of the hospitals subsequently approved conducting this clinical trial according to local legislation. The study was conducted in compliance with the provisions of the Declaration of Helsinki and ISO 14155:2011 Clinical investigation of medical devices for human subjects—Good clinical practice. All patients provided written informed consent. The study was registered on ClinicalTrials.gov (NCT01796236).

## Author Contributions

MH, SW, JI, MF, MJ, MM, HA, LA, and RS: study design. MH, JH, JT, JB, DM, SB, AR, and MC: conduct. MH, SW, JI, MJ, MM, and HA: analyses. MH, SW, JI, MJ, and RS: manuscript writing. MH, SW, JI, JH, JT, JB, DM, SB, AR, JM, MJ, and RS: manuscript revisions/review. All: final approval.

### Conflict of Interest

This research was sponsored and funded by Cochlear Bone Anchored Solutions AB in full. SW, MF, and JI are paid employees of Cochlear Bone Anchored Solutions AB. MH declares a travel grant of Cochlear Bone Anchored Solutions AB. MM and HA are a paid consultant to Cochlear Bone Anchored Solutions AB. The remaining authors declare that the research was conducted in the absence of any commercial or financial relationships that could be construed as a potential conflict of interest.

## References

[1] World Health Organization WHO Global Estimates on Prevalence of Hearing Loss. Geneva: World Health Organization (2012).

[2] Mathers CD Loncar D. Projections of global mortality and burden of disease from 2002 to 2030. PLoS Med. (2006) 3:2011–30. 10.1371/journal.pmed.0030442 17132052 PMC1664601

[3] Mohr PE Feldman JJ Dunbar JL McConkey-Robbins A Niparko JK Rittenhouse RK Skinner MW. The societal costs of severe to profoundhearing loss in the United States. Int J Technol Assess Health Care. (2000) 16:1120–35. 10.1017/S0266462300103162 11155832

[4] Tambs K. Moderate effects of hearing loss on mental health and subjective well-being: results from the Nord-Trøndelag Hearing Loss Study. Psychosom Med. (2004) 66:776–82. 10.1097/01.psy.0000133328.03596.fb 15385706

[5] Grutters JPC Joore MA Van Der Horst F Verschuure H Dreschler WA Anteunis LJC. Choosing between measures: comparison of EQ-5D, HUI2 and HUI3 in persons with hearing complaints. Qual Life Res. (2007) 16:1439–49. 10.1007/s11136-007-9237-x 17647093 PMC2039846

[6] Mylanus EA van der Pouw KC Snik AF Cremers CW. Intraindividual comparison of the bone-anchored hearing aid and air-conduction hearing aids. Arch Otolaryngol Head Neck Surg. (1998) 124:271–6. 10.1001/archotol.124.3.271 9525510

[7] Yung M. Long-term results of ossiculoplasty: reasons for surgical failure. Otol Neurotol. (2006) 27:20–6. 10.1097/01.mao.0000176173.94764.f5 16371842

[8] Evans AK Kazahaya K Canal atresia: “Surgery or implantable hearing devices? The expert's question is revisited.” Int J Pediatr Otorhinolaryngol. (2007) 71:367–74. 10.1016/j.ijporl.2006.09.003 17196671

[9] Tjellström A Lindström J Hallén O Albrektsson T Brånemark P. Osseointegrated titanium implants in the temporal bone. A clinical study on bone-anchored hearing aids. Am J Otol. (1981) 2:304–10. 6894824

[10] Brånemark P-I Breine U Adell R Hansson BO Lindström J Ohlsson Å. Intra-osseous anchorage of dental prostheses:i. experimental studies. Scand J Plast Reconstr Surg Hand Surg. (1969) 3:81–100. 10.3109/02844316909036699 4924041

[11] Pfiffner F Caversaccio M Kompis M Comparisons of sound processors based on osseointegrated implants in patients with conductive or mixed hearing loss. Otol Neurotol. (2011) 32:728–35. 10.1097/MAO.0b013e31821a02dd 21646934

[12] Stenfelt S. Transcranial attenuation of bone-conducted sound when stimulation is at the mastoid and at the bone conduction hearing aid position. Otol Neurotol. (2012) 33:105–14. 10.1097/MAO.0b013e31823e28ab 22193619

[13] Kitterick PT Smith SN Lucas L. Hearing instruments for unilateral severe-to-profound sensorineural hearing loss in adults: a systematic review and meta-analysis. Ear Hear. (2016) 37:495–507. 10.1097/AUD.0000000000000313 27232073 PMC4998125

[14] Hol MKS Bosman AJ Snik AFM Mylanus EAM Cremers CWRJ. Bone-anchored hearing aid in unilateral inner ear deafness: a study of 20 patients. Audiol Neurootol. (2004) 9:274–81. 10.1159/000080227 15316200

[15] Draaijers LJ Tempelman FRH Botman Y a M Tuinebreijer WE Middelkoop E Kreis RW van Zuijlen PPM. The patient and observer scar assessment scale: a reliable and feasible tool for scar evaluation. Plast Reconstr Surg. (2004) 113:1960–5; discussion: 1966–7. 10.1097/01.PRS.0000122207.28773.56 15253184

[16] Siau D Dhillon B Andrews R Green KMJ. Bone-anchored hearing aids and unilateral sensorineural hearing loss: why do patients reject them? J Laryngol Otol. (2015) 129:321–5. 10.1017/S0022215115000602 25776860

[17] Snik A Mylanus E Proops D Wolfaardt J Hodgetts W Somers T. Consensus statements on the BAHA system: where do we stand at present? Ann Otol Rhinol Laryngol Suppl. (2005) 195:2–12. 10.1177/0003489405114S1201 16619473

[18] Badran K Arya AK Bunstone D Mackinnon N. Long-term complications of bone-anchored hearing aids: a 14-year experience. J Laryngol Otol. (2009) 123:170. 10.1017/S0022215108002521 18492306

[19] Hultcrantz M. Outcome of the bone-anchored hearing aid procedure without skin thinning: a prospective clinical trial. Otol Neurotol. (2011) 32:1134–9. 10.1097/MAO.0b013e31822a1c47 21817939

[20] Holgers KM Tjellström A Bjursten LM Erlandsson BE. Soft tissue reactions around percutaneous implants: a clinical study of soft tissue conditions around skin-penetrating titanium implants for bone-anchored hearing aids. Am J Otol. (1988) 9:56–9. 3364537

[21] Larsson A Wigren S Andersson M Ekeroth G Flynn M Nannmark U. Histologic evaluation of soft tissue integration of experimental abutments for bone anchored hearing implants using surgery without soft tissue reduction. Otol Neurotol. (2012) 33:1445–51. 10.1097/MAO.0b013e318268d4e0 22918110

[22] van Hoof M Wigren S Duimel H Savelkoul PHM Flynn M Stokroos RJ. Can the hydroxyapatite-coated skin-penetrating abutment for bone conduction hearing implants integrate with the surrounding skin? Front Surg. (2015) 2:45. 10.3389/fsurg.2015.00045 26442276 PMC4568398

[23] van Hoof M Wigren S Blechert JI Flynn MC Eeg-Olofsson M Stalfors J Clinical outcomes of soft tissue preservation surgery with hydroxyapatite- coated abutments compared to traditional percutaneous bone conduction hearing implant surgery—a pragmatic multi-center randomized controlled trial. Front Surg. (2020) 7:5 10.3389/fsurg.2020.00005 PMC7066494 32211417

[24] Guyatt GH Oxman AD Kunz R Jaeschke R Helfand M Liberati A GRADE: incorporating considerations of resources use into grading recommendations. BMJ. (2008) 336:1170–3. 10.1136/bmj.39504.506319.80 18497416 PMC2394579

[25] Jaeschke R Guyatt GH Dellinger P Schünemann H Levy MM Kunz R. Use of GRADE grid to reach decisions on clinical practice guidelines when consensus is elusive. BMJ. (2008) 337:a744. 10.1136/bmj.a744 18669566

[26] Mauskopf JA Paul JE Grant DM Stergachis A. The role of cost-consequence analysis in healthcare decision-making. Pharmacoeconomics. (1998) 13:277–88. 10.2165/00019053-199813030-00002 10178653

[27] Cox RM Alexander GC. The abbreviated profile of hearing aid benefit. Ear Hear. (1995) 16:176–86. 10.1097/00003446-199504000-00005 7789669

[28] Feeny D Furlong W Torrance G Goldsmith C Zhu Z DePauw S. Multiattribute and single-attribute utility functions for the health utilities index mark 3 system. Med Care. (2002) 40:113–28. 10.1097/00005650-200202000-00006 11802084

[29] Longworth L Yang Y Young T Mulhern B Hernández Alava M Mukuria C. Use of generic and condition-specific measures of health-related quality of life in NICE decision-making: a systematic review, statistical modelling and survey. Health Technol Assess. (2014) 18:1–224. 10.3310/hta18090 24524660 PMC4780954

[30] Health Utilities Incorporated Summary Statistics for HUI Reference Scores of Health-Related Quality of Life. (2000). Available online at: http://www.healthutilities.com/49-HUI3Usa_F&M53$+$.pdf

[31] Horsman J Furlong W Feeny D Torrance G. The Health Utilities Index (HUI): concepts, measurement properties and applications. Health Qual Life Outcomes. (2003) 1:54. 10.1186/1477-7525-1-54 14613568 PMC293474

[32] Oppong R Jowett S Roberts TE. Economic evaluation alongside multinational studies: a systematic review of empirical studies. PLoS ONE. (2015) 10:e0131949. 10.1371/journal.pone.0131949 26121465 PMC4488296

[33] Nelissen RC den Besten CA Mylanus EAM Hol MKS Stability, survival, and tolerability of a 4.5-mm-wide bone-anchored hearing implant: 6-month data from a randomized controlled clinical trial. Eur Arch Oto Rhino Laryngol. (2016) 273:105–11. 10.1007/s00405-015-3593-x PMC4705128 25790770

[34] Faber HT Nelissen RC Kramer SE Cremers CWRJ Snik AFM Hol MKS. Bone-anchored hearing implants in single-sided deafness patients: long-term use and satisfaction by gender. Laryngoscope. (2015) 125:2790–5. 10.1002/lary.25423 26152833

[35] Morris D Hol M Ray J Toner J Hodgetts B Which device - when and why? The controversial role of bone conduction hearing devices in the rehabilitation of unilateral sensorineural hearing loss. J Laryngol Otol. (2016) 130:S121–2. 10.1017/S0022215116004308

[36] Desmet J Wouters K Bodt M Heyning P. Long-term subjective benefit with a bone conduction implant sound processor in 44 patients with single-sided deafness. Otol Neurotol. (2014) 35:1017–25. 10.1097/MAO.0000000000000297 24751733

[37] Colquitt JL Jones J Harris P Loveman E Bird A Clegg AJ. Bone-anchored hearing aids (BAHAs) for people who are bilaterally deaf: a systematic review and economic evaluation. Health Technol Assess. (2011) 15:1–200. 10.3310/hta15260 21729632 PMC4781533

[38] Crowson MG Tucci DL. Mini Review of the cost-effectiveness of unilateral osseointegrated implants in adults: possibly cost-effective for the correct indication. Audiol Neurotol. (2016) 21:69–71. 10.1159/000443629 26895350

[39] McLarnon C Davison T Johnson I. Bone-anchored hearing aid: comparison of benefit by patient subgroups. Laryngoscope. (2004) 114:942–4. 10.1097/00005537-200405000-00030 15126761

[40] Reinfeldt S Håkansson B Taghavi H Eeg-Olofsson M. New developments in bone-conduction hearing implants: a review. Med Devices. (2015) 8:79–93. 10.2147/MDER.S39691 25653565 PMC4303401

[41] Watson GJ Silva S Lawless T Harling JL Sheehan PZ. Bone anchored hearing aids: a preliminary assessment of the impact on outpatients and cost when rehabilitating hearing in chronic suppurative otitis media. Clin Otolaryngol. (2008) 33:338–42. 10.1111/j.1749-4486.2008.01698.x 18983343

[42] Ryan M. Discrete choice experiments in health care. BMJ. (2004) 328:360–1. 10.1136/bmj.328.7436.360 14962852 PMC341374

[43] Nussbaum MC Sen A The Quality of Life/Edited by Martha Nussbaum and Amartya Sen. (1993). Available online at: http://search.ebscohost.com/login.aspx?direct=true&db=edshlc&AN=edshlc.002500960-5&site=eds-live&scope=site

[44] Al-Janabi H Flynn T Coast J. Development of a self-report measure of capability wellbeing for adults: the ICECAP-A. Qual Life Res. (2012) 21:167–76. 10.1007/s11136-011-9927-2 21598064 PMC3254872

[45] Gluth M Eager K Eikelboom R Atlas M. Long-term benefit perception, complications, and device malfunction rate of bone-anchored hearing aid implantation for profound unilateral sensorineural hearing loss. Otol Neurotol. (2010) 31:1427–34. 10.1097/MAO.0b013e3181f0c53e 20729779

[46] McPherson K International differences in medical care practices. Health Care Financ Rev. (1989) 1989:9. PMC4195144 10318366

[47] Akeroyd M Brennan-Jones C Suller S. Re: bone-anchored hearing aids for people with bilateral hearing impairment: a systematic review. Clin Otolaryngol. (2012) 37:77. 10.1111/j.1749-4486.2012.02429.x 22433144

[48] Yang Y Longworth L Brazier J. An assessment of validity and responsiveness of generic measures of health-related quality of life in hearing impairment. Qual Life Res. (2013) 22:2813–28. 10.1007/s11136-013-0417-6 23709096 PMC3853410

[49] Joore M Brunenberg D Zank H van der Stel H Anteunis L Boas G. Development of a questionnaire to measure hearing-related health state preferences framed in an overall health perspective. Int J Technol Assess Health Care. (2002) 18:528–39. 10.1017/S0266462302000375 12391946

