# Supplementary material for: A Multinational Cost-Consequence Analysis of a Bone Conduction Hearing Implant System—A Randomized Trial of a Conventional vs. a Less Invasive Treatment With New Abutment Technology
Source: Front Neurol. 2020 Mar 13;11:106. doi: 10.3389/fneur.2020.00106 (PMC7082879; doi:10.3389/fneur.2020.00106)
Supplement: Supplementary file 1 [file Table_1.DOCX]

**S1 Supplemental Materials and Methods**

**Price sources**

Prices were extracted from publically available databases [1] and one commercial database as shown in Supplemental S1 Table 1. When no database was available, the scientific literature and grey literature (e.g. health economic reports) was searched for relevant prices. As a last resort, a general internet search was performed. Catalogue prices for medical device products from Cochlear Bone Anchored Solutions AB (Mölnlycke, Sweden) were provided by this manufacturer based on a confidentiality agreement for the sole purpose of the calculation and interpretation of the analysis. These prices were censored from presentation as they were considered to be trade secrets. Medication prices were identified based on their Anatomical Therapeutic Chemical (ATC) codes.

|  | **General health care costs** | **Medication costs** | **Medical device costs** | **Other** |
| --- | --- | --- | --- | --- |
| **The Netherlands** | [2] | Medicijnkosten  https://www.medicijnkosten.nl | Cochlear Bone Anchored Solutions AB | Nederlandse zorgautoriteit  <https://www.nza.nl/regelgeving/tarieven/>  [3][4][5][6][7][8] |
| **Spain** | Oblikue Consulting [9] | Vidal Vademecum Spain  <https://www.vademecum.es>  Colegio Oficial de Farmacéuticos de Pontevedra | Cochlear Bone Anchored Solutions AB | [10] |
| **Sweden** | Swedish Association of Local Authorities and Regions  https://stat.skl.se/SASStoredProcess/guest | Dental and Pharmaceutical Benefits Agency  <http://www.tlv.se/beslut/sok/lakemedel/> | Cochlear Bone Anchored Solutions AB | Norrlandstingens regionförbund  http://www.norrlandstingen.se  The Swedish Confederation of Professional Associations  <http://www.saco.se/studieval--karriar/studieval/yrken-a-o/audionom/>  Wage statistics for the various professions and counties  http://www.lonestatistik.se/loner.asp/yrke/Sjukskoterska-1226  [11][12][13][14] |
| **France** | - | Gestion risques maladie  http://www.codage.ext.cnamts.fr/  Prix des medicaments  http://medicprix.sante.gouv.fr | Cochlear Bone Anchored Solutions AB | Orientation-education  http://www.orientation-education.com/article/top-10-des-meilleurs-metiers-en-2012?i=9949  [15][16][17][18][19] |
| **Supplemental S1 Table 1 Price source databases** | | | | |

**Face validity and reliability assessment of retrieved prices**

Price data was classified in 4 categories (Supplemental S1 Table 2) which were ordered using a gradual decrease in reliability and certainty. Every price in the database was individually scored based on this scoring system. The frequency of resource consumption was not taking into account.

| 1 | The cost is derived from a reliable source. The cost is directly applicable without further corrections or modifications. The source states the price year of the cost. The uncertainty associated with this cost in relation to the interpretation in this trial is low. |
| --- | --- |
| 2 | The cost is derived from a moderately reliable source. Known or unknown factors can be expected to influence the applicability of this costs. Corrections and or modifications to this cost might be necessary for the correct interpretation of the costs within the context of this trial (e.g. exclude a top-up from a pharmacy). The uncertainty associated with this cost in relation to the interpretation in this trial is moderate. |
| 3 | The cost is derived from a poor source, the reliability cannot be assessed, the cost is extrapolated or estimated from an associated or similar cost from a reliable source (e.g. cost in a different country). The uncertainty associated with this cost in relation to the interpretation in this trial is moderate to high. |
| 4 | No reliable source for the cost could be identified. The cost is estimated from unreliable sources (e.g. internet searches). The uncertainty associated with this cost is high. |
| Supplemental S1 Table 2. Reliability of cost data classification | |

**Price level**

The price level was set at 2015. Historical prices were converted to 2015 using the consumer price index (retrieved from <https://research.stlouisfed.org> on 9 September 2015) for every individual country. Currency conversion for Swedish cost prices was performed using the rate at 9 September 2015 (1 SEK = 0.106 Euro) which was retrieved with Google Finance (Alphabet Inc., Mountain View, USA).

**References**

1. Dongen MS Van. Websites reporting medicine prices : a comparative analysis. In: http://apps.who.int/medicinedocs/documents/s17978en/s17978en.pdf. 2010.

2. Hakkaart- van Roijen, L., Tan, S.S., Bouwmans C a. M. Handleiding voor kostenonderzoek, methoden en standaard kostprijzen voor economische evaluaties in de gezondheidszorg (Guidelines for Pharmacoeconomic Research in the Netherlands). College voor zorgverzekeringen (Health Care Insurance Board (CVZ)). Coll voor Zorgverzekeringen. 2011; 1–127. Available: https://www.zorginstituutnederland.nl/binaries/content/documents/zinl-www/documenten/publicaties/overige-publicaties/1007-handleiding-voor-kostenonderzoek/Handleiding+voor+kostenonderzoek.pdf

3. Linssen AM, Anteunis LJC, Joore MA. The Cost-Effectiveness of Different Hearing Screening Strategies for 50- to 70-Year-Old Adults: A Markov Model. Value Heal. 2015;18: 560–569. doi:10.1016/j.jval.2015.03.1789

4. Máxima Medisch Centrum. Passanten prijslijst DBC-zorgproducten 2015 [Internet]. 2015. Available: https://www.mmc.nl/content/download/95888/823332/file/Passanten - Internetprijslijst DBC-zorgproducten 01-2015 tm 12-2015 versie 2, 2015-07-13.pdf

5. Tan SS, Van Putten E, Nijdam WM, Hanssens P, Beute GN, Nowak PJ, et al. A microcosting study of microsurgery, LINAC radiosurgery, and gamma knife radiosurgery in meningioma patients. J Neurooncol. 2011;101: 237–245. doi:10.1007/s11060-010-0243-4

6. Linden. Real‑world cost‑effectiveness of cetuximab in locally advanced squamous cell carcinoma of the head and neck. Eur Arch Otorhinolaryngol. 2014;

7. Damen THC, Wei W, Mureau MAM, Tjong-Joe-Wai R, Hofer SOP, Essink-Bot ML, et al. Medium-term cost analysis of breast reconstructions in a single Dutch centre: A comparison of implants, implants preceded by tissue expansion, LD transpositions and DIEP flaps. J Plast Reconstr Aesthetic Surg. 2011;64: 1043–1055. doi:10.1016/j.bjps.2010.12.028

8. Streeklaboratorium Haarlem. Tarieven Streeklab - Eerstelijns diagnostiek. 2013; 1–4. Available: https://www.medischcentrumtecleeff.nl/uploads/documenten/tarievenlijst_streeklab_1_juli_2013.pdf

9. Gisbert R, Brosa M, Consulting O. Spanish health costs database 2011. 2015.

10. Almenar L, Díaz B, Quesada A, Crespo C, Martí B, Mealing S, et al. Cost-effectiveness analysis of cardiac resynchronization therapy in patients with NYHA I and NYHA II heart failure in Spain. Int J Technol Assess Health Care. 2013;29: 140–6. doi:10.1017/S0266462313000123

11. Persson J, Ferraz-Nunes J, Karlberg I. Economic burden of stroke in a large county in Sweden. BMC Health Serv Res. 2012;12: 341. doi:10.1186/1472-6963-12-341

12. Ellström M, Ferraz-Nunes J, Hahlin M, Olsson JH. A randomized trial with a cost-consequence analysis after laparoscopic and abdominal hysterectomy. Obstet Gynecol. 1998;91: 30–34. doi:10.1016/S0029-7844(97)00579-6

13. Jansson SA, Backman H, Stenling A, Lindberg A, Rönmark E, Lundbäck B. Health economic costs of COPD in Sweden by disease severity - Has it changed during a ten years period? Respir Med. 2013;107: 1931–1938. doi:10.1016/j.rmed.2013.07.012

14. Andersson F, Borg S, Jansson S-A, Jonsson A-C, ERICSSON ÅSA, Prütz C, et al. The costs of exacerbations in chronic obstructive pulmonary disease (COPD). Respir Med. Elsevier; 2002;96: 700–708.

15. Eltchaninoff H, Prat A, Gilard M, Leguerrier A, Blanchard D, Fournial G, et al. Transcatheter aortic valve implantation: Early results of the FRANCE (FRench Aortic National CoreValve and Edwards) registry. Eur Heart J. 2011;32: 191–197. doi:10.1093/eurheartj/ehq261

16. Klouche S, Sariali E, Mamoudy P. Total hip arthroplasty revision due to infection: a cost analysis approach. Orthop Traumatol Surg Res. 2010;96: 124–32. doi:10.1016/j.rcot.2010.02.005

17. Alzahouri K, Lejeune C, Woronoff-Lemsi MC, Arveux P, Guillemin F. Cost-effectiveness analysis of strategies introducing FDG-PET into the mediastinal staging of non-small-cell lung cancer from the French healthcare system perspective. Clin Radiol. 2005;60: 479–492. doi:10.1016/j.crad.2004.10.010

18. Depont F, Hunsche E, Abouelfath A, Diatta T, Addra I, Grelaud A, et al. Medical and non-medical direct costs of chronic low back pain in patients consulting primary care physicians in France. Fundam Clin Pharmacol. Wiley Online Library; 2010;24: 101–108.

19. Urdahl H, Knapp M, Edgell ET, Ghandi G, Haro JM. Unit costs in international economic evaluations: resource costing of the Schizophrenia Outpatient Health Outcomes Study. Acta Psychiatr Scand. Wiley Online Library; 2003;107: 41–47.
