# Supplementary material for: A Multinational Cost-Consequence Analysis of a Bone Conduction Hearing Implant System—A Randomized Trial of a Conventional vs. a Less Invasive Treatment With New Abutment Technology
Source: Front Neurol. 2020 Mar 13;11:106. doi: 10.3389/fneur.2020.00106 (PMC7082879; doi:10.3389/fneur.2020.00106)

**S2 Supplemental Results**

**Unit costing and the cost database**

From the Case Report Forms, 289 unique entries were retrieved from the ‘abutment change’, ‘extra visits’ and ‘concomitant treatment’ pages. These were clustered in 10 labels and reduced to a set of 126 unique and relevant items. The relevant medication contained 24 different ATC codes. Costs were identified in France, Spain, Sweden and the Netherlands, for respectively 2904, 3151, 2911 and 2012 different types, ways of administration and dosages of medication. These were matched to the collected items. In case several prices existed for different methods of treatment administration, brands or dosages (for pharmaceutical products) associated with an ATC code, a median was taken from a sub selection of options. This sub selection reflected the most common method(s) of use and administration as collected in this study and in general in case the specific information was missing, as judged by the first author (MvH). This resulted in a price for medication associated with a unit of daily use. The cost for the incidental units: ‘surgery – local anesthesia’, ‘surgery – general anesthesia’, ‘abutment change’, ‘local anesthetic injection’, ‘ciprofloxacin injection’, ‘healing cap with a gauze and ointment’ and ‘peri-abutment skin revision surgery’ were based on a combination of items. These items, except for those related to the primary surgery, were subsequently clustered under labels: ‘local non-surgical treatment’ (e.g. ointments, local injections with analgesics or corticosteroids), ‘systemic treatment’ (e.g. antibiotic usage, corticosteroid treatment), ‘pain medication’ (oral administration), ‘local revision surgery’ (peri-abutment skin revision surgery), ‘diagnostics’ (X-ray skull, bacterial culture), ‘abutment removals’ and ‘abutment changes’. The cost for the hourly rate of the audiologist, surgeon and nurse was calculated based on Dutch national guidelines[CVZ], if this was not explicitly supplied by an available source.

**Face validity of identified costs: reliability and uncertainty assessment**

In general, half of all prices can be considered to be moderately to fairly reliable per country (Supplemental S2 Figure 1). Only a minor portion of prices (<15%) was either unreliable or highly reliable. No large or structural differences existed in reliability between countries.

**Supplemental S2 Figure 1. Reliability and uncertainty assessment**

The percentage of the reliability and uncertainty classification (1 – most reliable; 4 – most unreliable – S1 Supplemental Materials and Methods Table 2) in the four countries for every individual cost. NL Netherlands. SE Sweden. ES Spain. FR France.


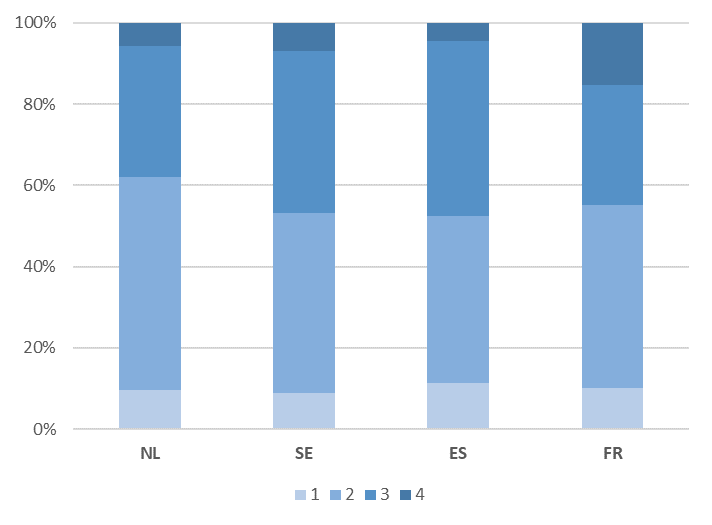

Supplement: Supplementary file 2 [file Table_2.DOCX]
